# Expressional variations of Kaiso: an association with pathological characteristics and field cancerization of OSCC

**DOI:** 10.1186/s12885-022-10014-7

**Published:** 2022-09-17

**Authors:** Shaheen Ahmed, Saeed Khan, Muhammad Asif Qureshi, Uzma Bukhari, Mehak Anis, Muhammad Nouman Mughal

**Affiliations:** 1 Department of Oral and Maxillofacial Surgery, Dow International Dental College, Dow University of Health Sciences, Karachi, Pakistan; 2 Department of Molecular Pathology, Dow International Medical College, Dow University of Health Sciences, Karachi, Pakistan; 3 Department of Pathology, Dow International Medical College, Dow University of Health Sciences, Karachi, Pakistan; 4 Department of Oral and Maxillofacial Surgery, Baqai Dental College, Baqai Medical University, Karachi, Pakistan; 5 Department of Surgery, Aga Khan Medical College, Aga Khan University, Karachi, Pakistan

**Keywords:** Kaiso, E-Cadherin, Oral cancer, Field cancerization, Oral squamous cell carcinoma, Immunohistochemistry (IHC)

## Abstract

**Background:**

A group of genetically altered cells that have not transformed into a clinical or histologically identifiable state of malignancy but contains a higher risk of transforming into one is known as the field of cancerization. Numerous molecules are being investigated for their significance in the development of this phenomenon. One such protein of this family is Kaiso also known as ZBTB33 (Zinc Finger and BTB Domain containing 33). This protein belongs to the POZ-ZF family of transcription factors and may have functional tasks similar to its other siblings such as the growth and development of vertebrates and the pathogenesis of neoplastic diseases. Nevertheless, its role in the pathogenesis, progression, epithelial mesenchyal transition and field cancerization in case of oral cancer still needs exploration. Hence, this study was designed to explore the expressional differences between the mucosa of controls and those diagnosed with oral squamous cell carcinoma (OSCC).

**Methods:**

Soft tissue samples were obtained from the main tumor, tumor periphery and opposite buccal mucosa of 50 oral cancer patients, whereas normal mucosa was taken from 50 volunteers undergoing elective tooth removal. The acquired samples were subjected to Immunohistochemical exploration for expression of Kaiso and E-Cadherin. The expression was measured using Image-J IHC profiler and summed as Optical density. The Optical density values were then subjected to statistical analysis.

**Results:**

Results revealed a significant differential expression of Kaiso between the mucosal tissues taken from oral cancer patients and controls (*p*-value: < 0.0001), showing almost 50% down-regulation of Kaiso in all three tissue samples taken from oral cancer patients as compared to normal mucosa.

**Conclusion:**

Kaiso has a significant difference of expression in the mucosa of oral cancer patients as compared to the mucosa of normal patients, making it a probable contributor to disease pathogenesis and field cancerization.

**Supplementary Information:**

The online version contains supplementary material available at 10.1186/s12885-022-10014-7.

## Background

Neoplasms arise due to compound genetic and epigenetic aberrations that consequentially transmute cells of a particular organ initiating an advanced invasive disease [1]. It is hypothesized that these genetic and epigenetic aberrations are not localized to a specific group of cells, rather they involve all the daughter cells residing in a particular field or surrounding tissues of the tumor [2]. Although not currently transformed completely in invasive disease, these resident cells harbour enough genetic anomalies which may transform them into malignant tumors at any given time in the future [2]. This populace of daughter cells residing in an organ, which has genetic aberrations but does not display morphological transformation consistent with malignant neoplasm, corroborates with the concept of field cancerization. This was initially presented in 1953 by Slaughter *et al* [3]. Scientific studies are being conducted to pinpoint molecular signatures which may help identify these genetically anomalous cell populations which have not adapted to the pathologic morphology of invasive carcinoma. When identified, these molecular markers may provide remarkable utility in terms of screening, diagnostics and targeted therapeutics. Some of the molecular markers found associated with oral field cancerization include cytokeratins 7, 8, 13, 16, and 19, [4] type 2 chain ABH antigen, [5] cyclin D1, [6, 7] EGFR, [8–10] TGF-α, [10] Ki-67, [11] BCL-2, [12] vascular markers (VwF, CD31, αVβ3, α-SMA), [13] and p53 [14–19]. One of the proteins of interest in this regard is Kaiso. Kaiso is a member of the broad-complex, tram track and bric-a-brac/poxvirus and zinc finger (BTB/POZ) family with a subfamily of zinc finger proteins (POZ-ZF) [20]. Transcription factors belonging to this family have been known to play a part in the growth and developmental aspects of vertebrates which indicates that Kaiso might have comparable functionality [21]. Kaiso’s functional preferences are described to change in context to its interactions with different proteins [22]. For instance, it has been demonstrated that Kaiso activates BCL-2 a protein that inhibits apoptotic death of the cell while deactivating the two pro-apoptotic proteins BAX and BIK hence leading to apoptotic diffidence [23]. Another example of context-specific functionality of Kaiso is demonstrated by its binding with wild type and mutated p53 where it promotes apoptosis when bonded with the former and suppresses programmed cell death when bonded with later, respectively [24, 25]. Considering the examples given previously Kaiso may be a vital part of pathogenic pathways leading to the development of neoplastic diseases. To date, ample scientific literature has verified Kaiso’s involvement in different types of cancers such as breast CA, [24, 26–29] Lung (NSCLC), [30] prostate CA, [31, 32] and pancreatic cancer (PDAC) [33]. It is also advocated that translocation of cytoplasmic fragment of E-cad into the nucleus modulates the activity of Kaiso, which exerts suppressive effects on the promoter region of target genes that are still mostly unidentified [34]. Having said that, depicting Kaiso as a tumor suppressor or tumor promoter with certainty. Furthermore, the scientific literature is also deficient regarding Kaiso’s functional role in the pathogenesis of oral mucosal cancer and devising Epithelial Mesenchymal Transition (EMT) has been a challenging task. It is yet to be explored whether the exposure of oral mucosa to known carcinogens such as tobacco, betel quid, betel nuts and other combination products has any effect on expressional values of Kaiso or not. Consequently, this study was directed at exploring expressional changes of Kaiso in mucosal specimens taken from the main tumor, the periphery of the tumor, and opposing mucosa of patients diagnosed with oral squamous cell carcinoma compared with subjects who were disease-free and were not exposed to any of the chemical carcinogens associated with the disease.

## Methodology

### Study design and study setting

The study design was analytical cross-sectional. Total soft tissue specimens from 50 biopsies of proven oral squamous cell carcinoma (OSCC) patients and 50 control tissues were obtained from patients who were attending the Department of Oral and Maxillofacial Surgery at Dow International Dental College, Dow University of Health Sciences, Karachi, Pakistan. The samples were collected during patients’ therapeutic surgery from OSCC cases and during elective tooth removal from controls, respectively. Cognizant patient consent was obtained from all study participants and endorsement was taken from the Institutional Review Board of Dow University (IRB-1319/DUHS/Approval/2019). Patients’ biographic information, medical history, extent and characteristics of the disease were documented in pre-designed proformas. Small tissue samples were taken from the Tumor (Labeled T), the periphery of the tumor (Labelled P), and the opposing mucosa (Labeled O), of OSCC patients who matched the inclusion and exclusion criteria (Table 1). From controls who matched the inclusion and exclusion criteria (Table 1), a small tissue specimen was collected during elective surgical removal of wisdom teeth, labelled as C. All samples were immediately placed in 10% buffered Neutral formalin.

**Table 1:** Inclusion and exclusion criteria for cases and controls

**CASES**	**Inclusion Criteria**	**Exclusion Criteria**
	Biopsy proved cases of OSCC regardless of age/gender	-Recipients of prior chemotherapy or radiotherapy - Patients with any congenital syndrome, autoimmune diseases, chronic inflammatory diseases, and any other chronic illness - Poorly fixed tissue
**CONTROLS**	**Inclusion Criteria**	**Exclusion Criteria**
	Adult patients undergoing elective surgical tooth extractions for wisdom teeth Patients not exposed to any chemical carcinogens such as betel quid, betel nut, and any form of tobacco	-Patients with infected teeth -Patients with any congenital syndrome, autoimmune diseases, chronic inflammatory diseases, and neoplastic diseases -Patients with the habit of tobacco use in any form, Betel quid use, betel nut use, alcohol, or any combination of these products

### Sample size

The power of the test was calculated to justify the sample size of tissues per group using PASS version 15 software (NCSS, Kaysville, Utah, USA), based on a one-way analysis of variance test with 95% confidence of interval, an effect size of 0.854374 with 50 tissues in each of four groups computed using results from the expression of kaiso in mucosal samples taken from Tumor, Tumor’s periphery and Opposing cheek of OSCC patient. It was found to be more than 99%. The same power of the test was found using results from relationship between Kaiso and E-Cadherin with 95% confidence of interval, an effect size of 0.5537 with 50 tissues in each of four groups.

### Tissue processing and immunohistochemical staining

The tissues obtained were embedded in paraffin to form blocks. Ultra-thin consecutive sections of 4 µm were incised from every single block. To scrutinize histopathological physiognomies, a section from each sample was stained with Hematoxylin & Eosin stains. After analyzing histologic characteristics of the tissues, we probed them for expression of Kaiso and E-Cadherin using Immunohistochemical staining. Briefly, the tissue sections were deparaffinized by xylene, after which tissue hydration was done, followed by heating in the microwave with citrate buffer (pH = 6) for retrieval of antigens. The tissues were then subjected to incubation in hydrogen peroxide for 20 min to mollify endogenous peroxidase interactions. Subsequently, tissue specimens were exposed to 15 bovine serum albumin for an hour to terminate the non-specific binding of antibodies. Next, tissues were incubated with polyclonal rabbit anti-kaiso (Thermo Fischer Scientific; PA5-81,890) and monoclonal mouse anti-E-cadherin (Thermo Fischer Scientific; 4A2c7) antibodies which were prepared at a dilution of 1:50. Human gall bladder tissues were used as a positive control for anti-kaiso, whereas gastric cancer tissues were used as positive controls for anti-E-cadherin antibodies as described in the catalogue provided by the manufacturer. After incubation with the Kaiso and E-cadherin, antibody slides were washed using Phosphate Buffered Saline. Then, EnVision FLEX, High pH (Link) (DAKO, Agilent Tech.) was used for detecting primary antibodies. Finally, the tissues were incubated with diaminobenzidine (DAB) + chromogen followed by a hematoxylin stain. After completing the chemical treatment, tissue slides were mounted using DPX and coverslip.

### Imaging and expressional analysis

IHC slides from both cases and controls were photographed using Nikon Eclipse 80i at 40 × which are provided in the supplementary data upon reviewer’s request. To quantify the protein expression images were then analyzed using ImageJ IHC profiler software The software semi-quantifies the protein in the studied samples by assigning an automated score in a four tier system: high positive (HP), positive (P), low positive (LP) and negative (N). This is achieved by measuring pixel intensity that range from 0 to 255, where 0 represents the darkest shade and 255 represent the lightest shade of color as standard. The histogram profile zones are equally divided on the pixel color intensity bar. All intensities from 0 to 60 score for high positive zone, 61–120 for positive zone, 121 to 180 for low positive zone and 181 to 235 for negative zone. Values ranging from 235 to 255 represent fatty tissues and are excluded from the score determination zones. The four values obtained from ImageJ were then converted to optical density score, via formula given by S.Jafari et al. [34]:

$$(4\mathrm{HP}+3\mathrm{P}+2\mathrm{LP}+1\mathrm{N}) / 100$$

The optical density values were then used for statistical analysis.

### Statistical analysis

Data normality was analyzed using the Kolmogorov–Smirnov test. Differential expression of the Kaiso and E-cadherin protein among the cases and controls, different tumor sizes and tumor grades was analyzed using one-way ANOVA. Pearson’s r test was done to find the correlation between the Expression of Kaiso and all the histological features recorded. The statistical analysis was performed employing Graph Pad Prism software and a *p*-value of less than 0.05 was considered significant. Also, all the results were further analysed as (positive + low positive) values VS high positive values as per advice of the reviewer but no significant differences were present than the cumulative results obtained as optical density. Hence these have not been included in the manuscript, although the file is added to the supplementary material.

## Results

Out of total 50 cases 41 were obtained from males, whereas 9 were obtained from the females. The mean age of OSCC case group was 50.8 ± 11 years. Further stratification of the cases was done according to Broders grading System Table 2, and according to tumor size Table 3.

**Table 2:** Distribution of cases according to broders grading system [35]

Grade	No. of Cases	Percentage
Well Differentiated	18	36
Moderately Differentiated	20	40
Poorly Differentiated	12	24

**Table 3:** Distribution of cases according to tumor size

Tumor Size	No. of Cases	Percentage
T1	7	14
T2	9	18
T3	6	12
T4	28	56

### Differential analysis

Differential expression of Kaiso was assessed between tissue specimens taken from controls and tissue specimen taken from OSCC patients to determine expressional variations between the two and to assess if there is any kind of evidence present regarding field cancerization. Furthermore, expressional variations were assessed in different grades and sizes of tumors to determine Kaiso’s role in disease progression.

#### KAISO Expression in controls and tumor, periphery and opposing buccal mucosa of OSCC patients

**Fig. 1:**
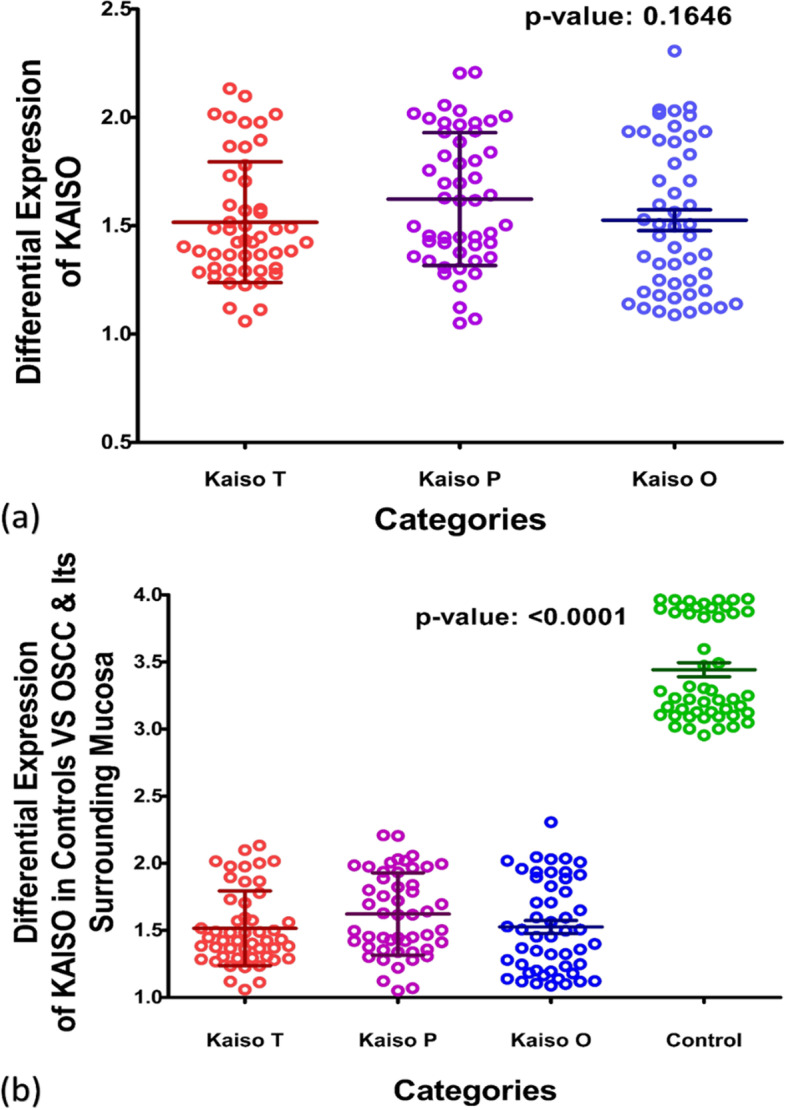
Differential Expression of Kaiso in OSCC (**a**) and Controls against OSCC (**b**). The graph in figure **a** demonstrate the difference of expression in Kaiso among tissue specimens taken from OSCC cases; whereas figure **b** demonstrates differential expression between the Controls and OSCC tissue specimens. All values plotted here are in the form of optical density (OD). The large central line represents mean, whereas small horizontal lines above and below represent the standard deviation

When expression of Kaiso was compared among the two groups specimens taken from tumor (T), periphery (P), and opposite buccal mucosa (O); no significant differential expression was seen among the three groups in OSCC cases of Kaiso (*p*-value 0.1646), Fig. 1(a). But when all three values were analyzed against the expressional values of Control group a significant difference of expression was observed between tissues of controls and cases with a *P*-value of < 0.0001, Fig. 1(b). Mean expression values of Kaiso protein in all specimen are given in Table 4 showing a drastic decrease in kaiso expression in all tissues obtained from OSCC group.

**Table 4:** Mean expression of kaiso in cases and controls

***Controls (C)***	***OSCC CASES***			
	Tumor (T)	Periphery (P)	Opposite (O)	*P*-Value
*3.443* ± *0.3743*	1.516 ± 0.2790	1.623 ± 0.3068	1.526 ± 0.3397	< 0.0001

#### E-Cadherin expression in controls and tumor, periphery and opposing buccal mucosa of OSCC patients

**Fig. 2:**
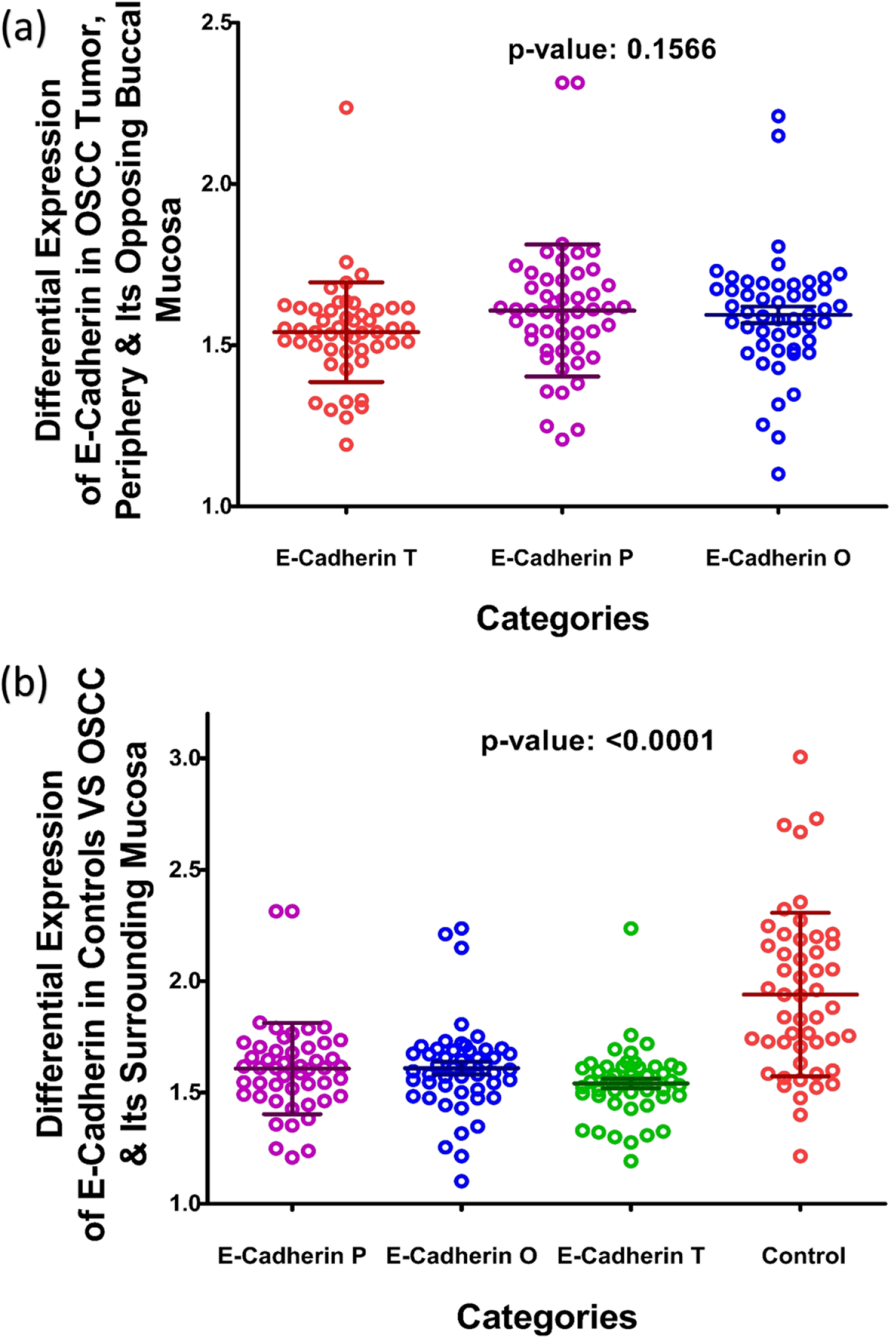
Differential Expression of E-Cadherin in OSCC (**a**) and Controls against OSCC (**b**). The graph in figure **a** demonstrate the difference of expression in E-Cadherin among tissue specimens taken from OSCC cases; whereas figure **b** demonstrates differential expression between the Controls and OSCC tissue specimens. All values plotted here are in the form of optical density (OD). The large central line represents mean, whereas small horizontal lines above and below represent the standard deviation

When expression of E-Cadherin was compared among the two groups specimens taken from tumor (T), periphery (P), and opposite buccal mucosa (O); no significant differential expression of E-Cadherin was seen among the three groups in OSCC cases (*p*-value 0.1566), Fig. 2(a). But when all three values were analyzed against the expressional values of Control group a significant difference of expression was observed between tissues of controls and cases with a *P*-value of < 0.0001, Fig. 2(b). Mean expression values of E-Cadherin protein in all specimen are given in Table 5 showing a drastic decrease in E-Cadherin expression in all tissues obtained from OSCC group as compared to Controls (*P*-value: < 0.0001).

**Table 5:** Mean expression of e-cadherin in cases and controls

***Controls (C)***	***OSCC CASES***			
	Tumor (T)	Periphery (P)	Opposite (O)	*P*-Value
*1.940* ± *0.3671*	1.541 ± 0.1546	1.608 ± 0.2059	1.609 ± 0.3397	< 0.0001

#### KAISO expression in different grades and sizes of OSCC

**Fig. 3:**
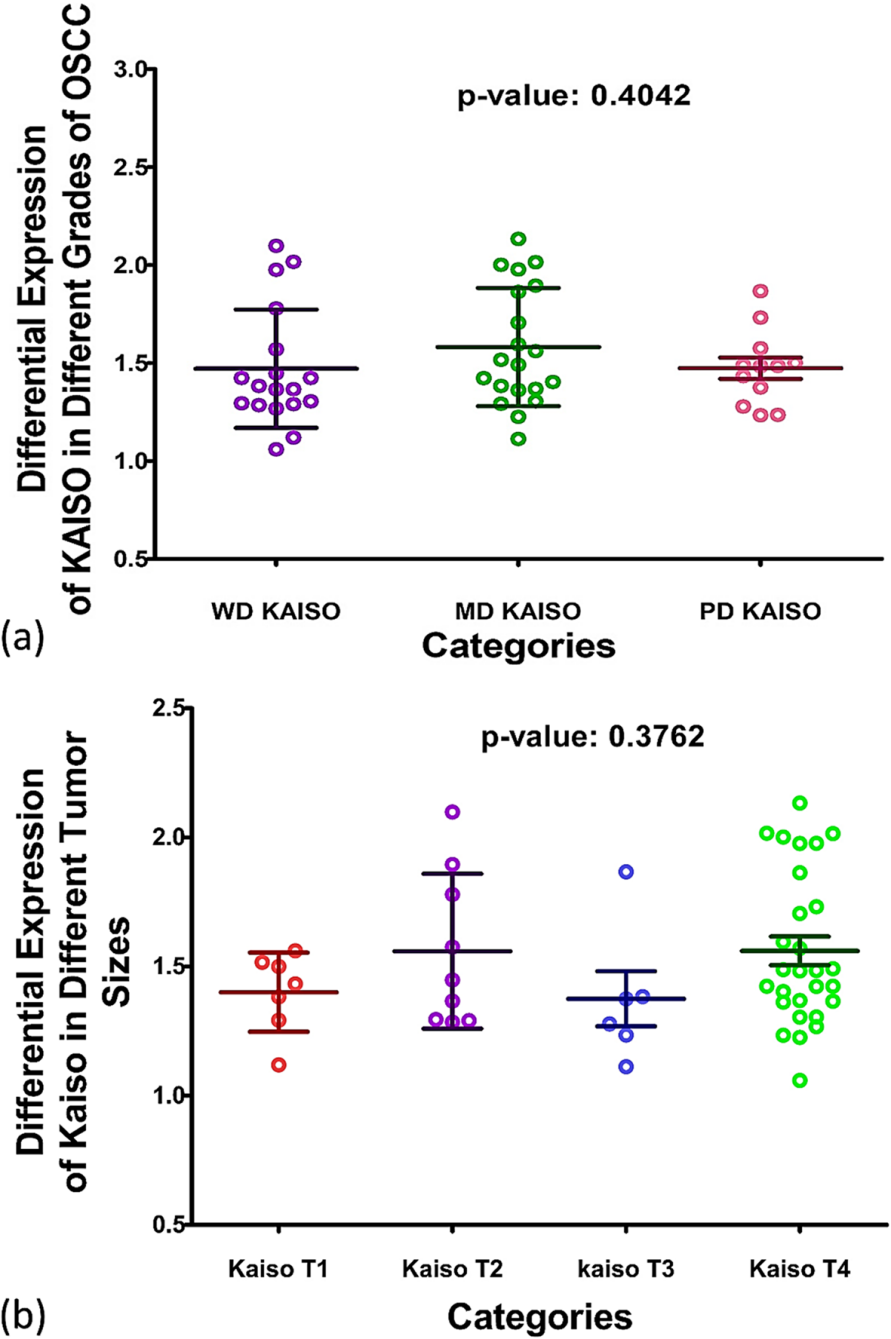
Differential Expression of Kaiso in Different Tumor Grades (**a**) and Tumor Sizes (**b**). The graph in figure **a** demonstrate the difference of expression in Kaiso among different Tumor Grades; whereas figure **b** demonstrates differential expression of Kaiso among different tumor sizes. All values plotted here are in the form of optical density (OD). The large central line represents mean, whereas small horizontal lines above and below represent the standard deviation

On comparison between the different grades of tumor, no significant alteration could be observed among the mean expression values of Kaiso among different grades of OSCC, as confirmed by *p*-value 0.4042, Fig. 3(a). Similarly, no significant mean expressional variation could be found in different tumor (T) sizes (*p*3-value: 0.3762), Fig. 3(b). Mean expression values of Kaiso in different grades and T sizes are given in Table 6.

**Table 6:** Mean expression of kaiso in different tumor grades and sizes

**Kaiso Expression in Different Grades of OSCC**			
**Well Differentiated OSCC**	**Moderately Differentiated OSCC**		**Poorly Differentiated OSCC**
**1.471 ± 0.3016**	1.582 ± 0.3016		1.474 ± 0.1899
**Kaiso Expression in Different Tumor Size**			
**T1**	**T2**	**T3**	**T4**
**1.401 ± 0.1532**	1.559 ± 0.2998	1.375 ± 0.2607	1.561 ± 0.2938

### Correlation analysis

**Fig. 4:**
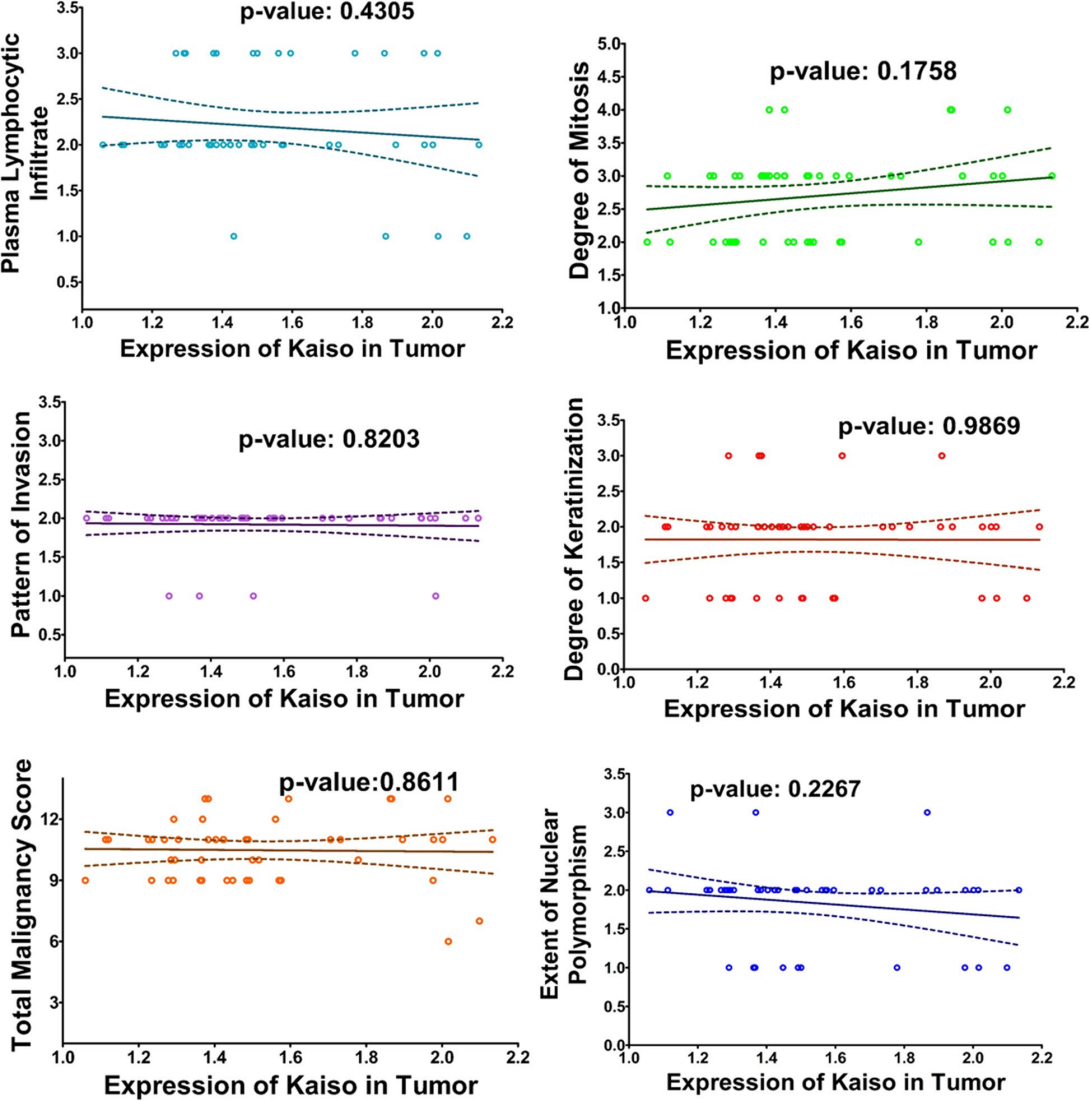
Correlation between Expression of Kaiso and Histological Feature Scores from Bryne’s TIF Grading System. The graphs here demonstrate the correlation between expression of Kaiso in OSCC compiled as Optical Density and scores given to each histological feature according to Bryne’s scoring system. Each histological feature is correlated separately followed by the correlation with the total malignancy score

**Fig. 5:**
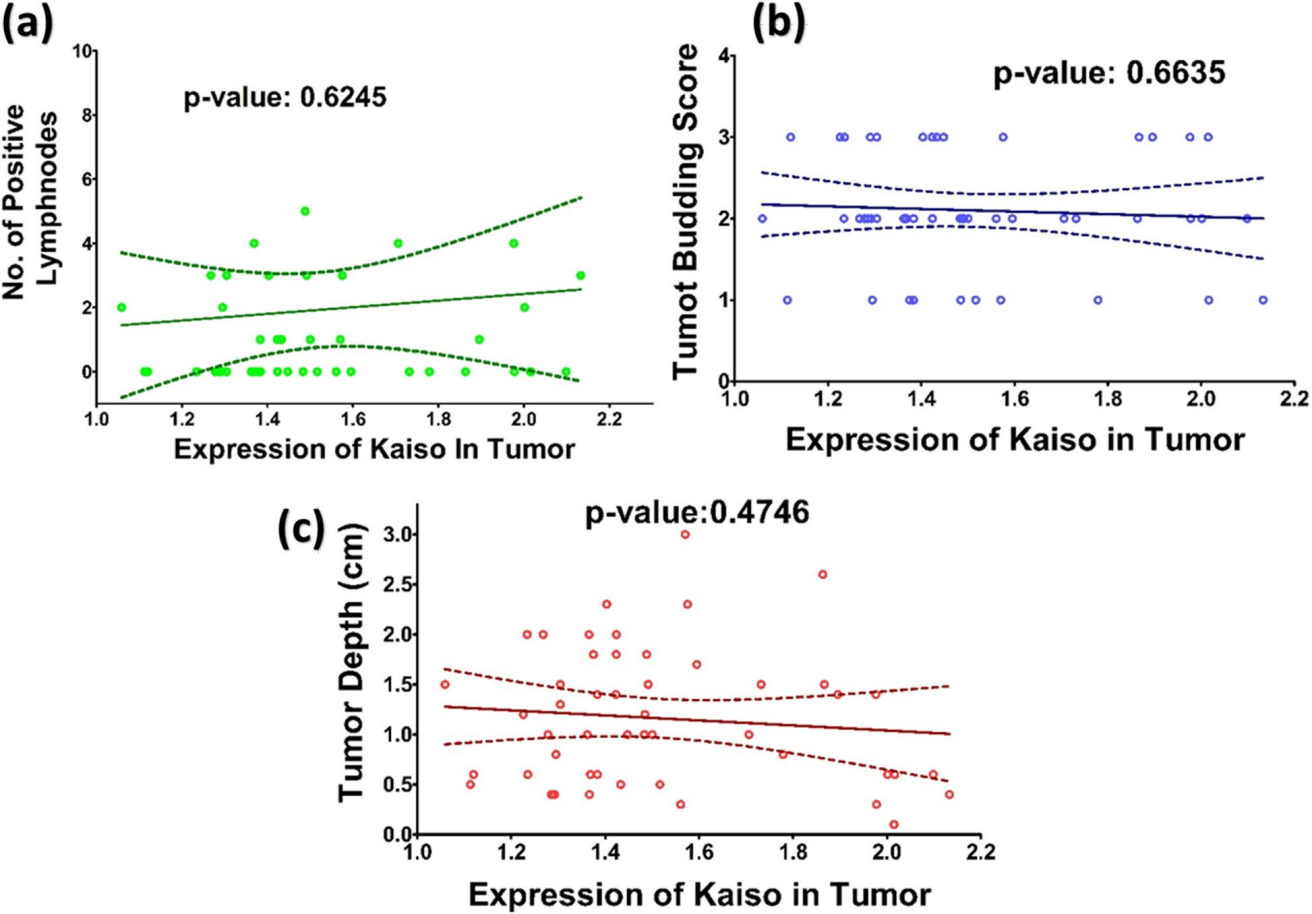
Correlation between Expression of Kaiso with Tumor Positive Lymphnodes (**a**), Tumor Depth (**b**) and Tumor Budding Score (**c**). The graphs here demonstrate expression of Kaiso in OSCC compiled as Optical Density, correlated with number of tumor positive lymphnodes, tumor depth measured in centimeters and tumor budding score

**Fig. 6:**
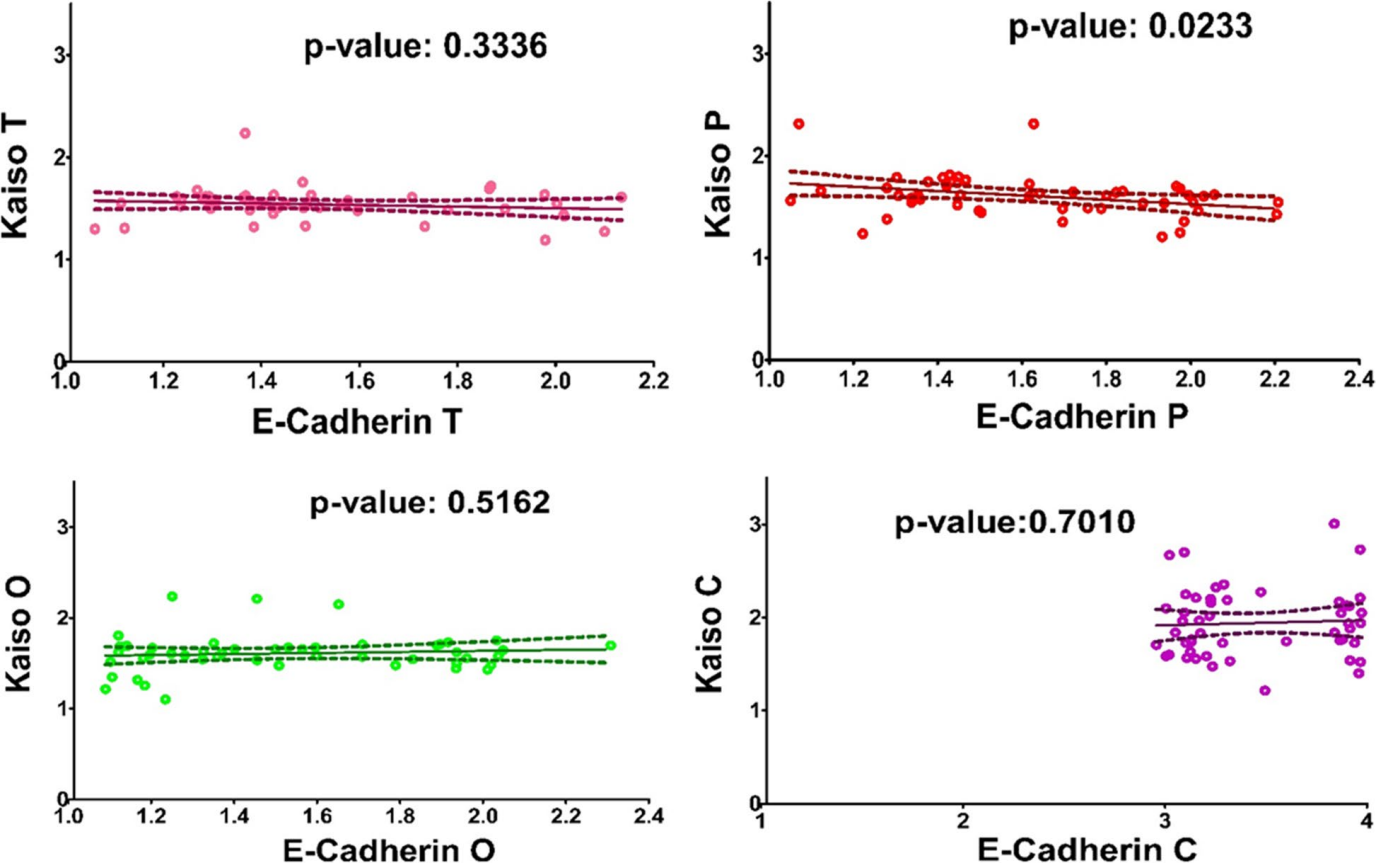
Correlation between Expressions of Kaiso with E-Cadherin. The graphs here demonstrate expression of Kaiso correlated with expression of E-Cadherin in OSCC (T, P, O) and Controls

Expression of kaiso was correlated with various histological features such as histological features defined in bryne’s TIF scoring system [36] and also total malignancy score (Table 7); Fig. 4, number of positive lymph nodes Fig. 5(a), tumor depth Fig. 5(b), and tumor budding scores [37] (Table 8), Fig. 5(c). No significant positive or negative correlation could be found or any of the physio-pathological characteristics as depicted by the straight correlation lines of the graphs and *p*-values mentioned in the respective figures. Similarly, Expression of Kaiso was correlated with E-Cadherin, Fig. 6. The only significant correlation could be observed between the two is in the region of tumor periphery. No other significant correlations could be observed.

**Table 7:** Bryne’s Tumor Invasive Front (TIF) Grading System [36]

Morphological feature	SCORE			
	1	2	3	4
**Degree of keratinization**	Highly keratinized (> 50% of the cells)	Moderately keratinized (20- 50% of the cells)	Minimal keratinization (5-20% of the cells)	No keratinization (0-50/, of the cells)
**Nuclear polymorphism**	Little nuclear polymorphism (> 75% mature cells)	Moderately abundant nuclear polymorphism (50-75% mature cells)	Abundant nuclear polymorphism (25- 50% mature cells)	Extreme nuclear polymorphism (0- 25% mature cells)
Number of mitoses (high power field)*	0-1	2-3	4-5	> 5
**Pattern of invasion**	Pushing, well delineated infiltrating borders	Infiltrating, solid cords, bands and/ or strands	Small groups or cords of infiltrating cells (*n* > 15)	Marked and widespread cellular dissociation in small groups and/ or in single cells (*n* < 15)
**Lymphoplasmacytic infiltrate**	Marked	Moderate	Slight	None

**Table 8:** Tumor budding score [37]

No. of Tumor Buds per 10 *HPF × 40	Score
0 Tumor Buds = No Budding	1
1–14 tumor buds = low Budding	2
> 15 tumor buds = high Budding	3

^*^HPF High Powered Field

## Discussion

The concept of field cancerization was first suggested by Slaughter et al. after extensive histological examination of 783 cases of oral cancer, where he observed multiple centers of abnormal epithelium beyond the boundaries of the clinically evident malignant tumor. He observed that there was evidence of multiple microscopic foci of abnormal epithelial cells which later enlarge and coalesce to form a clinical picture of invasive carcinoma [3]. Later on, the term “Field Cancerization” was coined for this phenomenon and extensive scientific investigations on the molecular level were started regarding this concept to determine subsequent genetic aberrations, which is the driving force toward the malignant transformation of cells. Several genes and their expressional abnormalities have been implied as markers for the identification of field cancerization. These include, tumor suppressor genes such as p53, [38–40] p16, [41] Cyclin D1, [42] p21, [40]; Proto-oncogenes like, Ras [42] and Erb1 [43]; growth factor receptors such as EGFR [44], VEGF [45], TGF-α [10], and CD34 [46]. In this study, we have made an effort to determine whether expressional dysregulation of Kaiso has any role in field cancerization.

Kaiso is a member of the BTB/POZ-ZF family of site-specific transcription factors. The functional activity of a transcription factor has been considered vacillating due to its inclination towards context-specific suppression and/or activation of various genes in different types of cells [22]. Various factors are anticipated as game changers in Kaiso’s functional specificity towards a tumor suppressor or activator role. One of the factors may be Kaiso’s ability to bind both methylated and non-methylated sequence-specific sites (bi-modal DNA binding) [47, 48]. the second factor that should be taken into account is its SUMOylation which may play a role in the bimodal functional abilities of the proteins [49]. Another factor that can be taken into account is whether Kaiso binds with wild-type p53 or mutated p53 which determines the final functional path chosen by the protein [24, 25]. In this study we compared the expression of Kaiso in the mucosa of subjects who were never exposed to chemical carcinogens known to predispose to oral cancer, with the expression of Kaiso in biopsy-proven tumors and seemingly normal mucosa taken from the tumor periphery and opposing cheek of the same patient. The idea was to determine what path this protein takes in case of oral squamous cell carcinoma in terms of expression. Astonishingly, the findings show an almost 50% decreased expression of Kaiso in the mucosa of OSCC patients in comparison to controls with a *p*-value of < 0.0001 (Fig. 1b). Also, it can be appreciated that pure tumor tissue, peripheral mucosa and opposing mucosa of OSCC cases, all show similar mean expressions of Kaiso (Table 4), indicating a decreased Kaiso expression in the entire oral cavity and not specifically the tumor. These findings highlight that Kaiso might have a vital role in defining the field of cancerization in cases of oral squamous cell carcinoma. Furthermore, levels of E-cadherin expressions in the same tissue specimens of OSCC was also found significantly down regulated (Fig. 2b, Table 5) as compared to controls but when the two proteins were correlated with each other, no significant correlation could be observed between the two except in the region of tumor periphery (Fig. 6), where a negative correlation could be observed between the two proteins. Also, a correlation analysis was done between the expressional variations of Kaiso with different tumor grades and tumor sizes which turned out to be insignificant (Fig. 3a and b). These outcome indicates that Kaiso’s expressional vicissitudes are more likely involved in the incidence of OSCC rather than its progression. The other inference that could be obtained from this study was that, although E-cadherin might be an independent marker of epithelial mesenchymal transition and field cancerization but it does not show any significant correlation with the expressional changes of kaiso in case if OSCC. Moreover, if E-cadherin affects kaiso in any way it is not in terms of expressional changes, rather it may affect Kaiso’s ability to alter the expression of target genes after binding with p120 as suggested by Van Roy et al [20]. Additionally, correlation analyses were done employing Kaiso expression in tumor tissues and the histological features of these specimens which are frequently used in terms of prognostic determinants of OSCC (Figs. 4 and 5), namely the number of positive lymph nodes, depth of tumor, number of tumor buds, degree of keratinization, number of mitotic figures, nuclear polymorphism, the pattern of invasion and extent of plasma-lymphocytic infiltrate. No significant correlation could be observed between the expression of Kaiso and histological features mentioned, which further strengthens our impression of Kaiso acting as a match stick to a haystack in the case of OSCC. A study by Cofre et al. demonstrated that diminution of Kaiso expression resulted in the augmented proliferation and reduced expression of differentiation markers; supporting our findings [50]. In contrast to this study there are several studies which have reported a higher expression of Kaiso associated with different types and pathological features of cancers. For instance, Pierre et al*.* reported a greater Kaiso expression in primary & metastatic tumor tissue specimens of colon, in comparison to normal [51]. Whereas, Jones et al*.* observed a higher Kaiso expression in malignant tumors of the prostate than benign prostatic hyperplasia. He also reported that higher expression of Kaiso had a correlation with higher tumor grade [31]. In another scientific paper, Jones et al. reported similar associations between high Kaiso expression in pancreatic ductal carcinomas and higher tumor grades and sizes [33]. Analogous findings are reported in the case of breast carcinoma in various studies [5, 7, 22, 23]. Differences which might be responsible for such contrasting results might include exposure to chemical carcinogens specific to the oral cavity such as betel nut and betel quid promoting a factor specific functioning of Kaiso.

What mechanism and/or factors are behind this striking decrease in Kaiso’s expression in the mucosa of oral cancer patients and whether Kaiso plays a role in devising epithelial mesenchymal transition in OSCC still needs to be explored, giving us a future direction for a much needed scientific research.

## Conclusion

In conclusion, it can be safely assumed that Kaiso has a role in the genesis of Oral cancer indicated by its expressional dysregulation in OSCC patients. Kaiso may also serve as a marker for field cancerization in OSCC patients. These findings make Kaiso a suitable candidate for targeted therapy. Hence, it is recommended that other aspects of Kaiso’s functionality should be assessed to further elaborate its role in the pathogenesis of oral squamous cell carcinoma and field cancerization.

### Limitations of the study

Like most studies, there were some limitations to this study as well. The measurement of protein was done by one method only. This may be performed with other methods of protein estimation to cross-check the results. No specimens were included from patients with precancerous conditions, the inclusion of which may help determine a threshold level of Kaiso that indicates the conversion of a premalignant state into a malignant state before the appearance of physiopathological characteristics. It is therefore required that the research is validated with improved sample size and multicenter study, with populations from different ethnicities, and with groups including oral premalignant states and lesions in future. Furthermore, survival analysis of the study could not be performed as the sample collection for this study was started in year 2019 (IRB ref no: IRB-1319/DUHS/Approval/2019) and continued till 2021, the patients are still being followed for survival data which will be published it in near future.

## Supplementary Information

**Additional file 1.**

## Data Availability

The data collected and used for this research is the property of Dow University of Health Sciences, whereas the data used in this study is available upon request from the corresponding author.

## Abbreviations

OSCC: Oral Squamous Cell Carcinoma
IHC: Immunohistochemistry
PBS: Phosphate Buffered Saline
BNF: Neutral Buffered Formalin
