# Supplementary material for: Expressional variations of Kaiso: an association with pathological characteristics and field cancerization of OSCC
Source: BMC Cancer. 2022 Sep 17;22:990. doi: 10.1186/s12885-022-10014-7 (PMC9482199; doi:10.1186/s12885-022-10014-7)

Controls

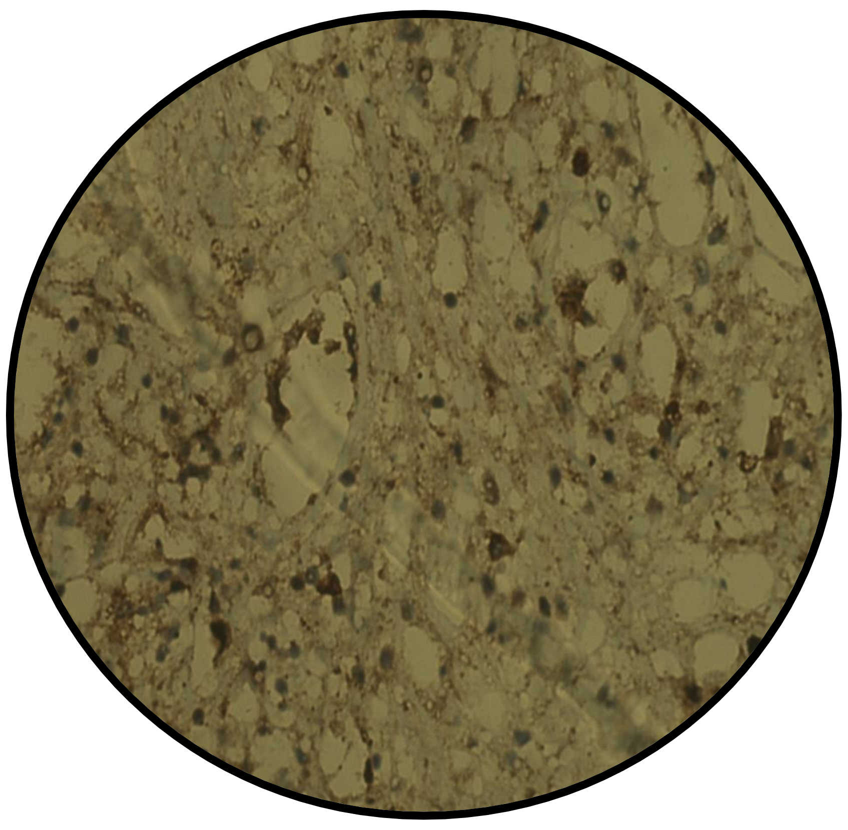

C1

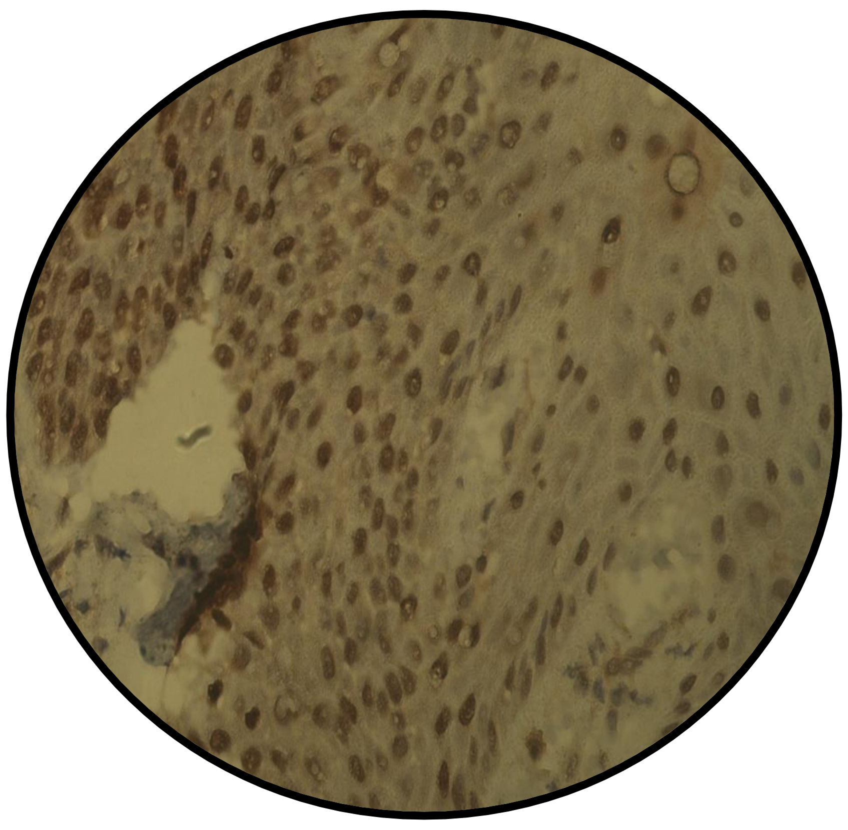

C2

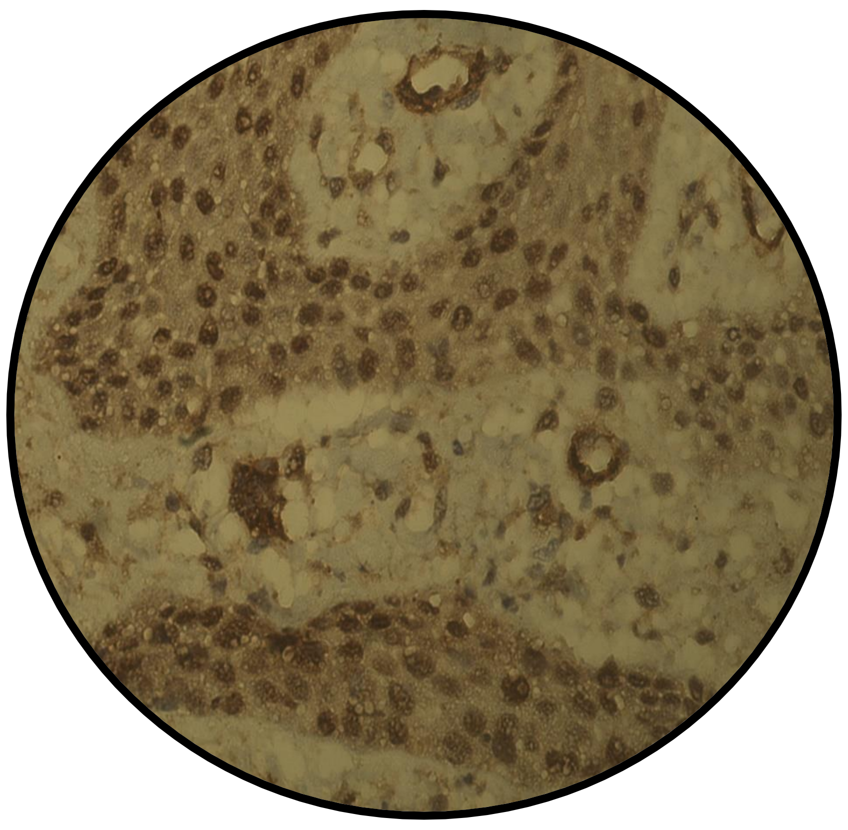

C3

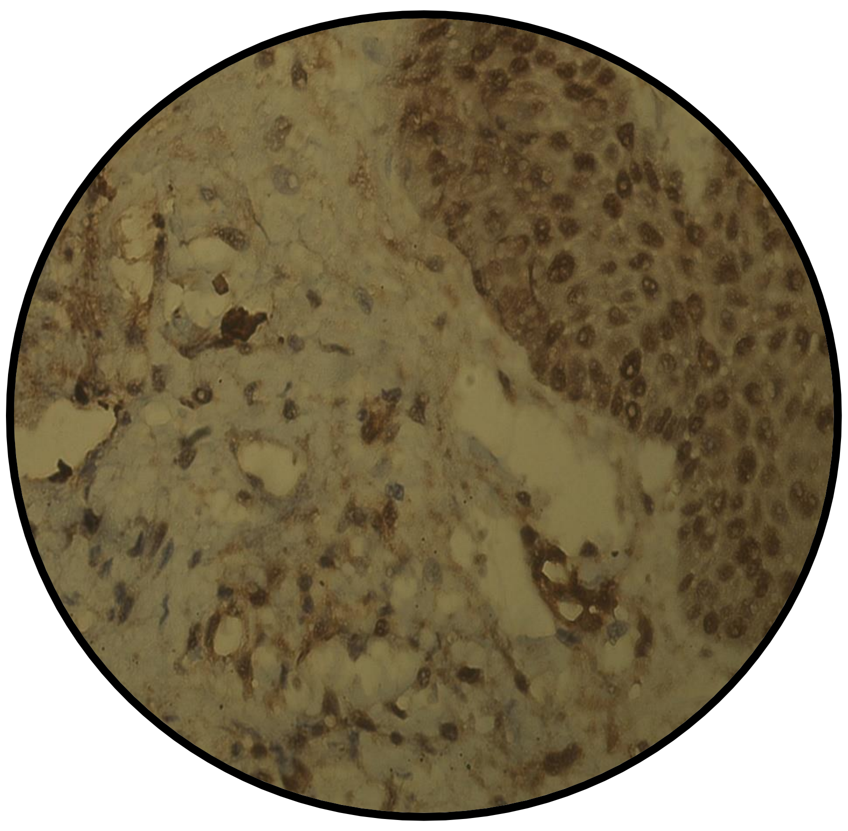

C4

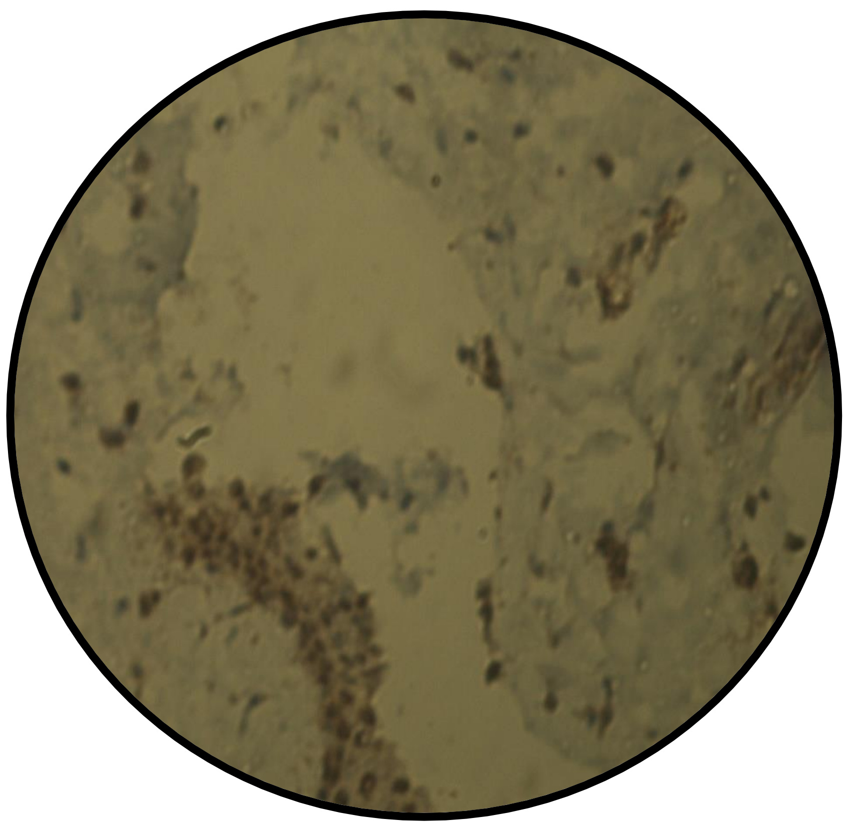

C5

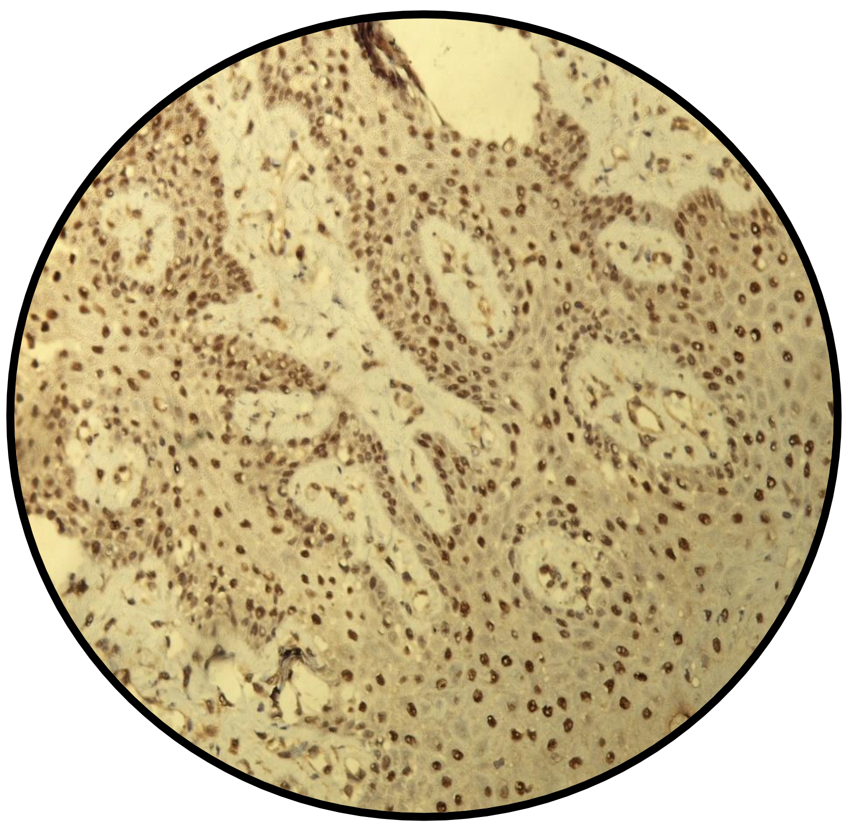

C6

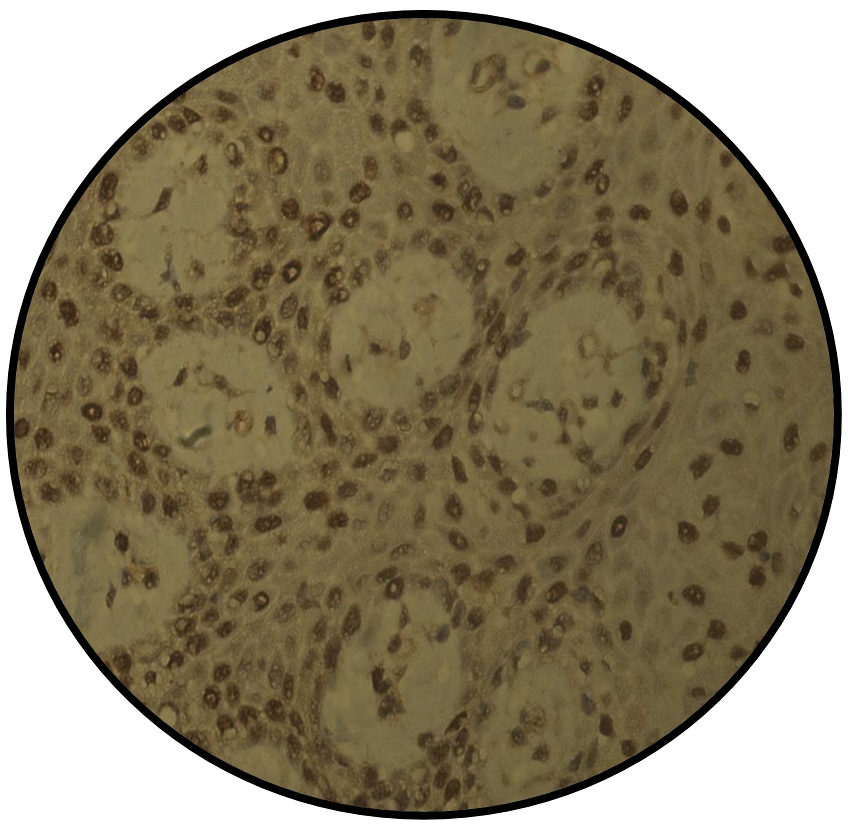

C7

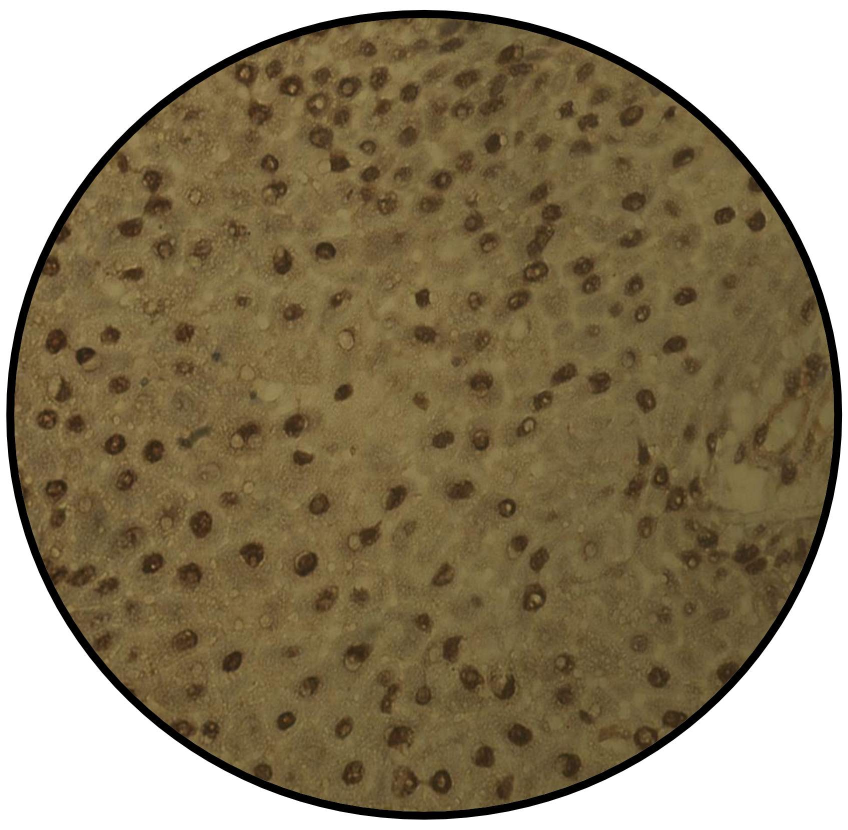

C8

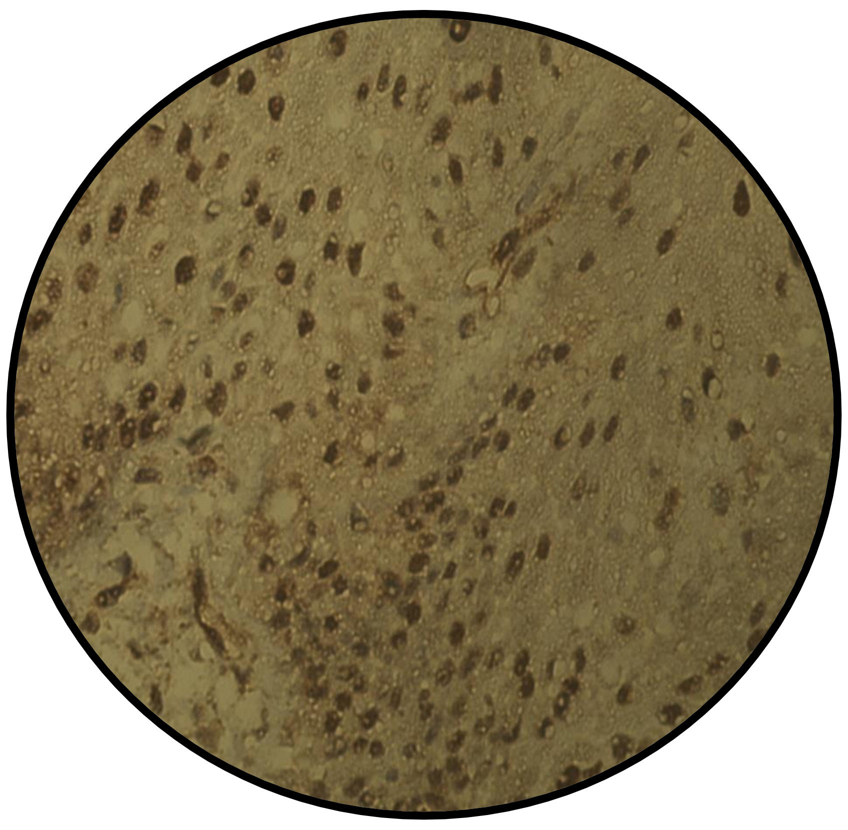

C9

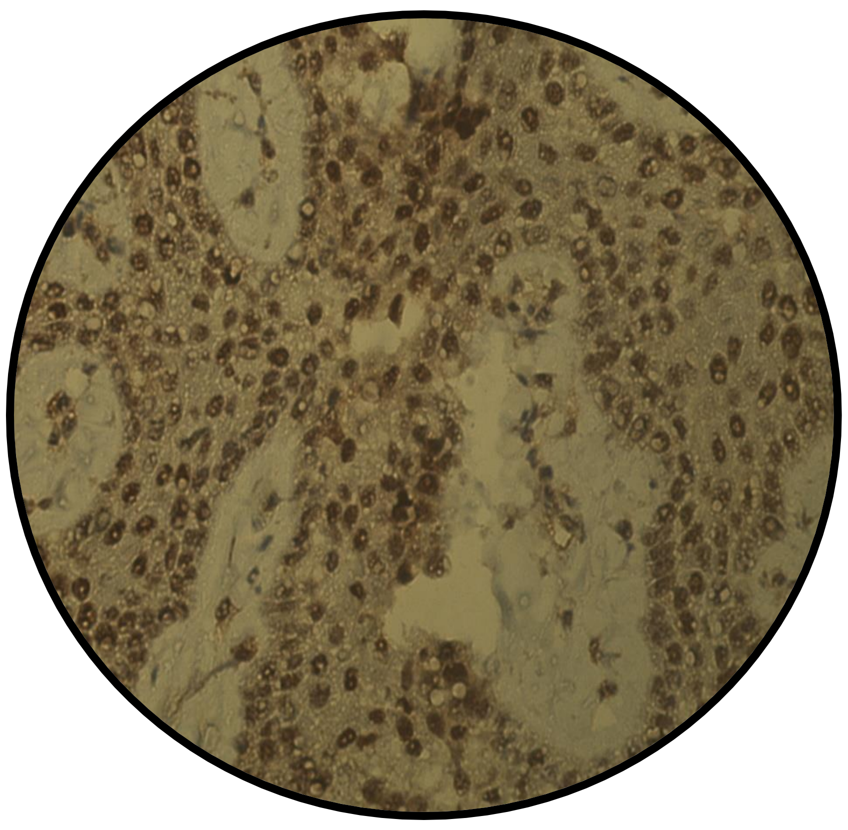

C10

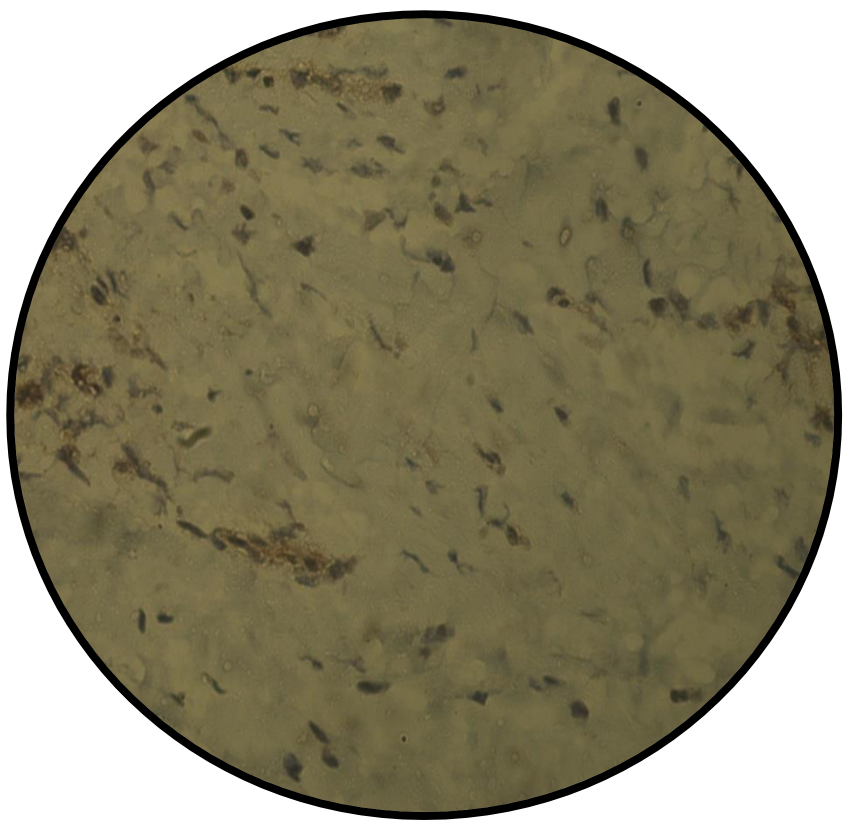

C11

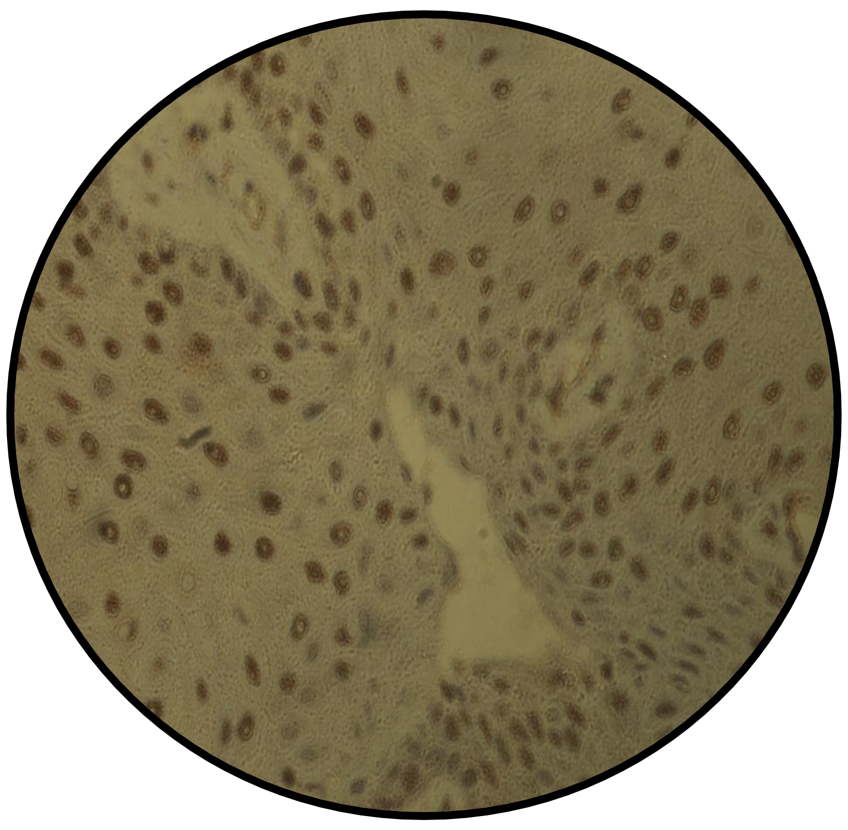

C12

Controls

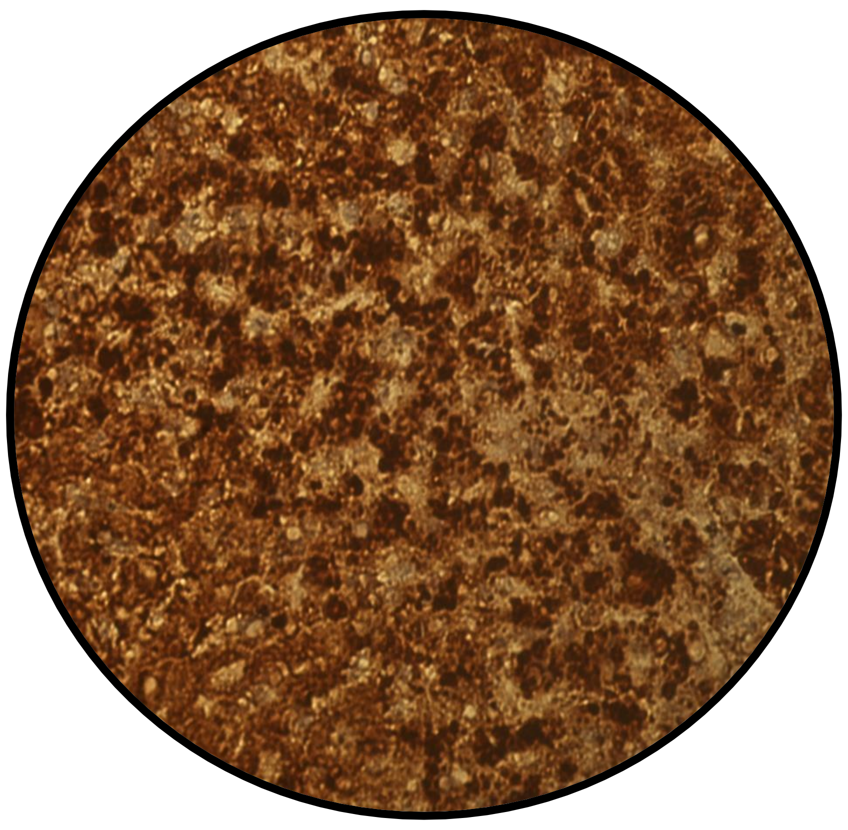

C13

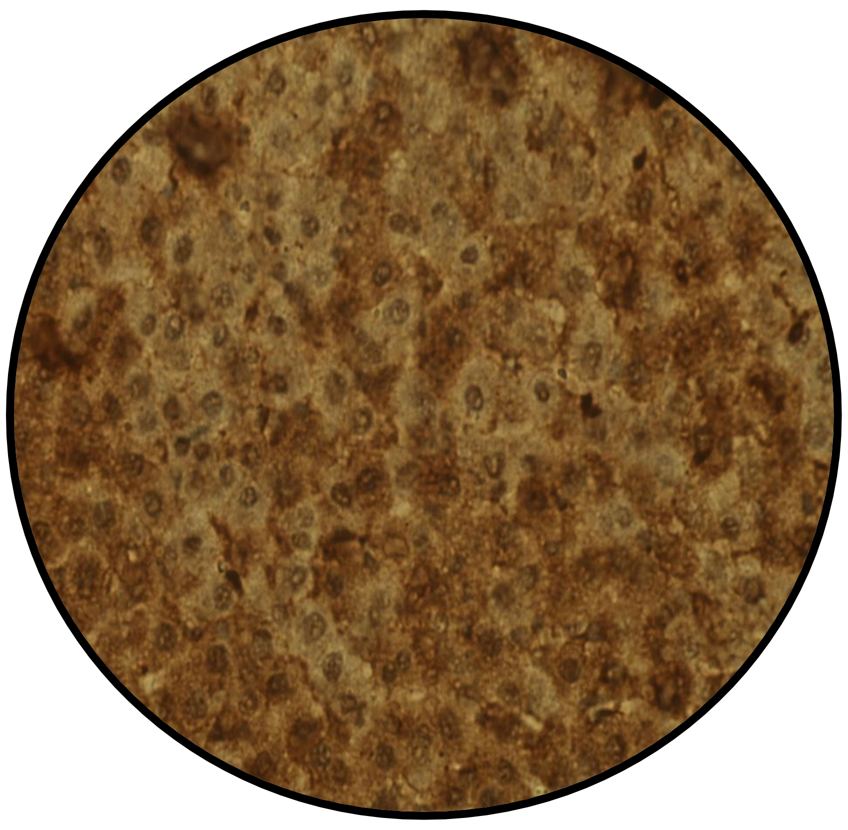

C14

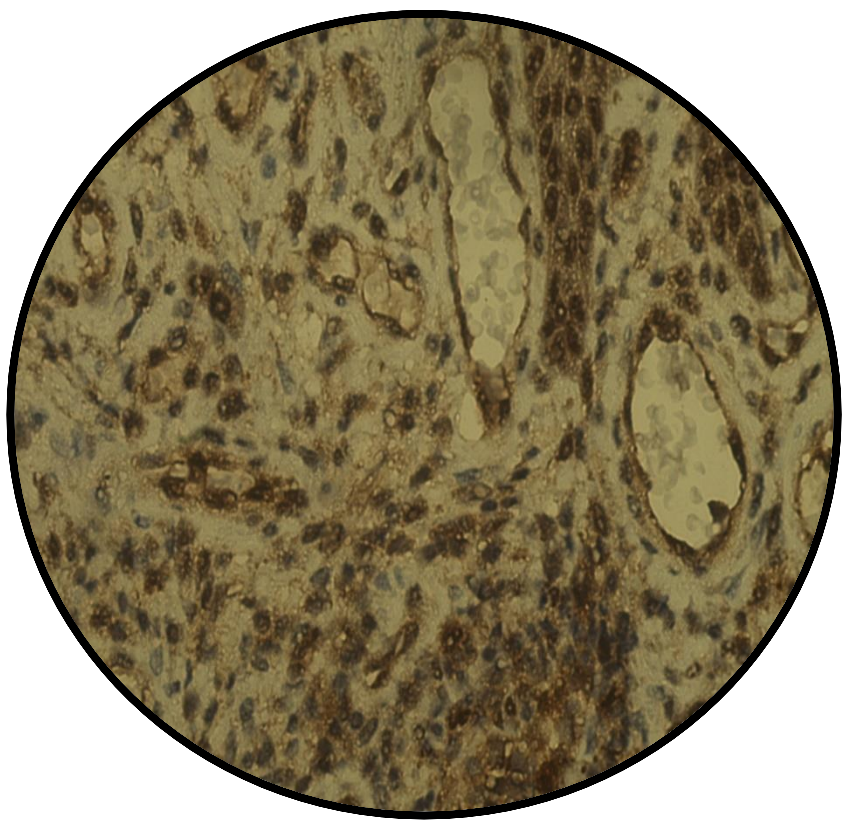

C15

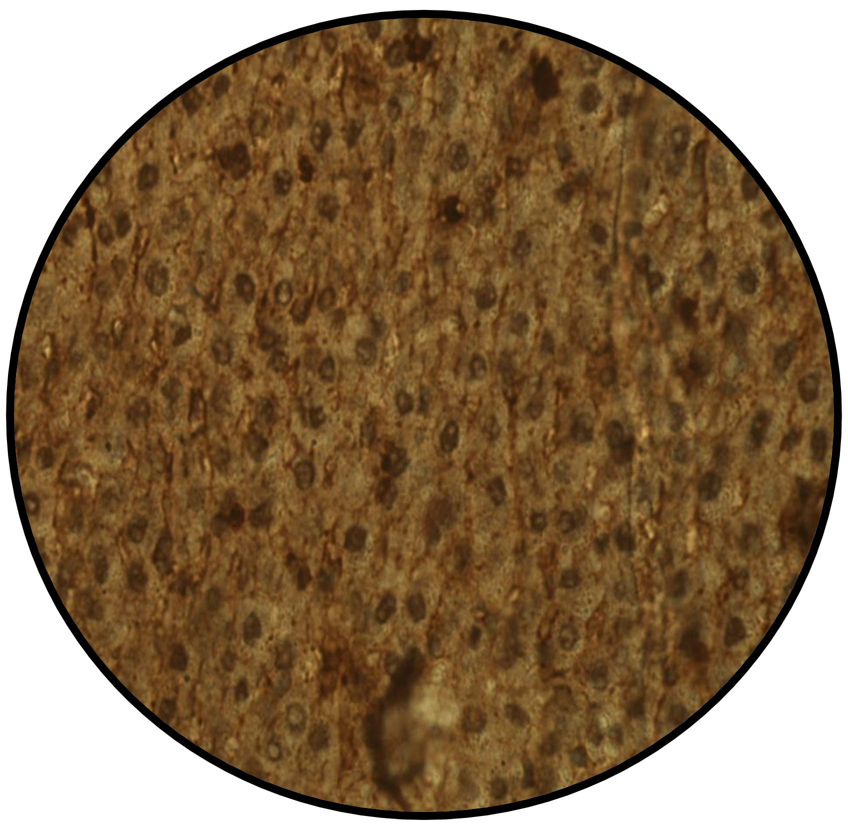

C16

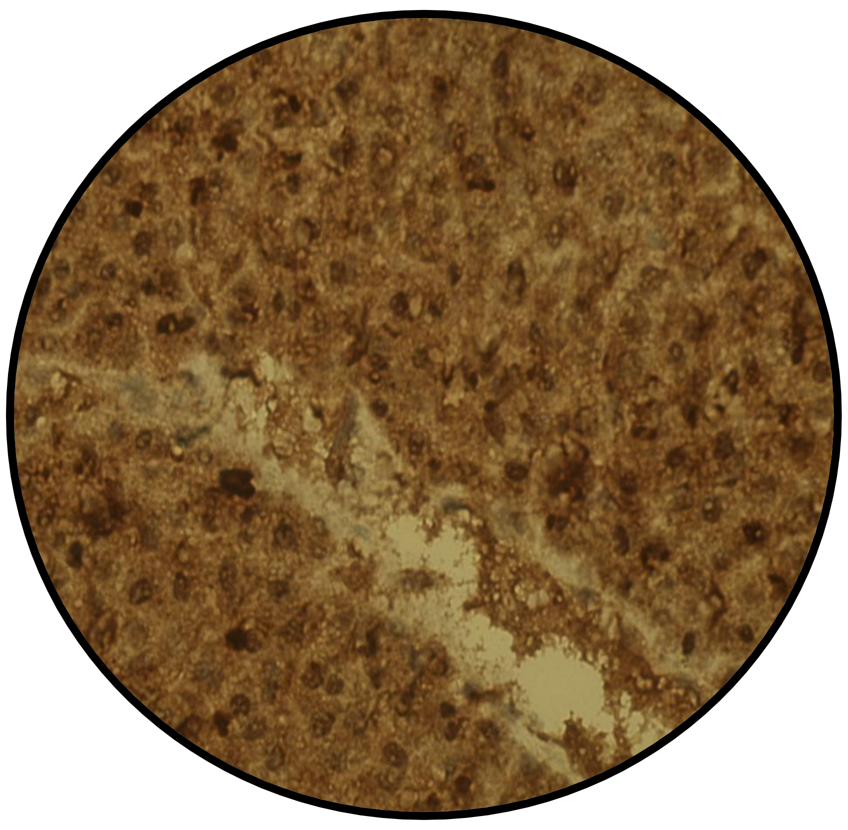

C17

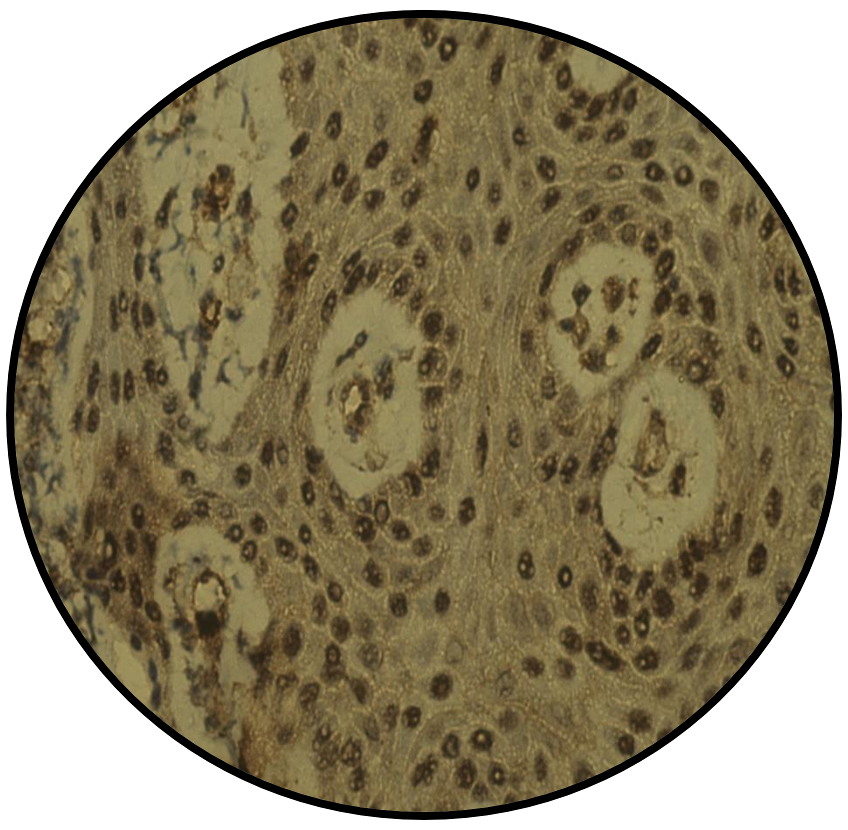

C18

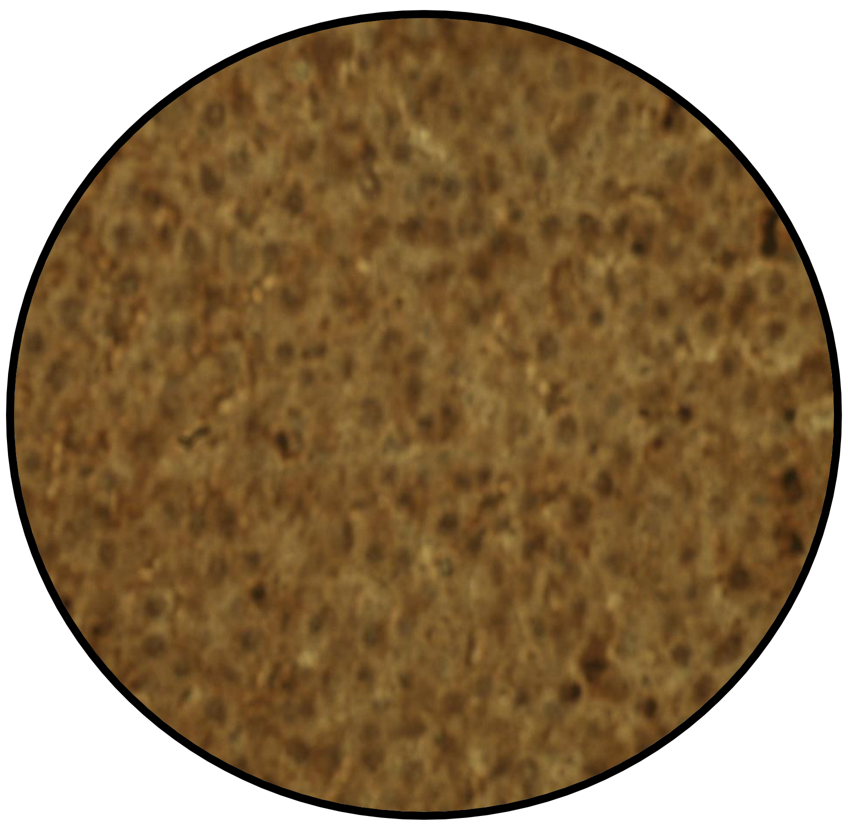

C19

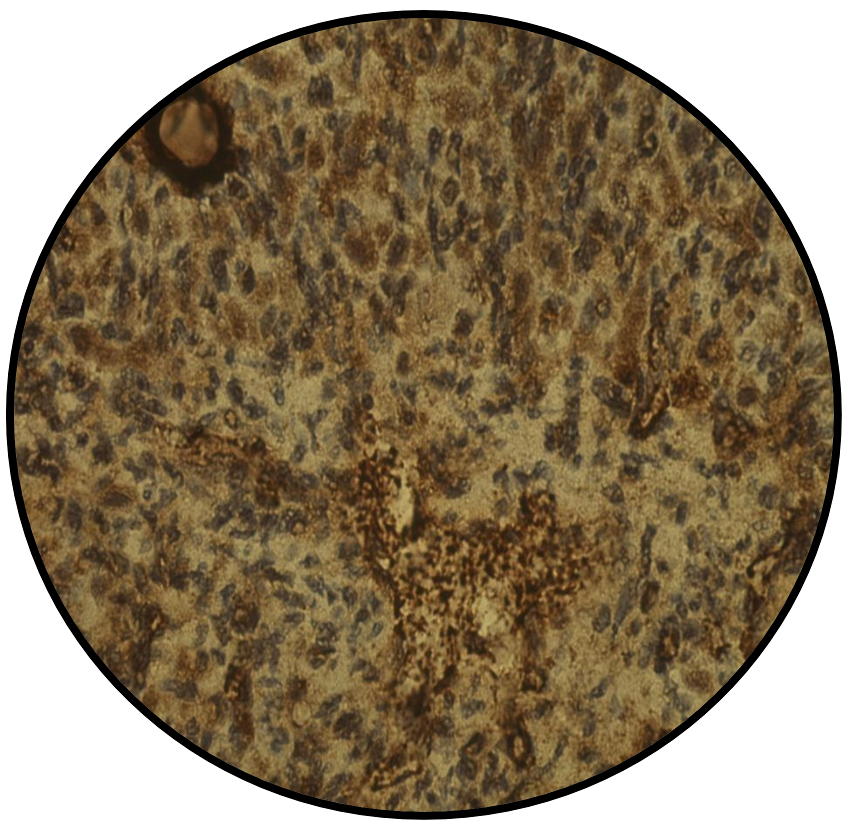

C20

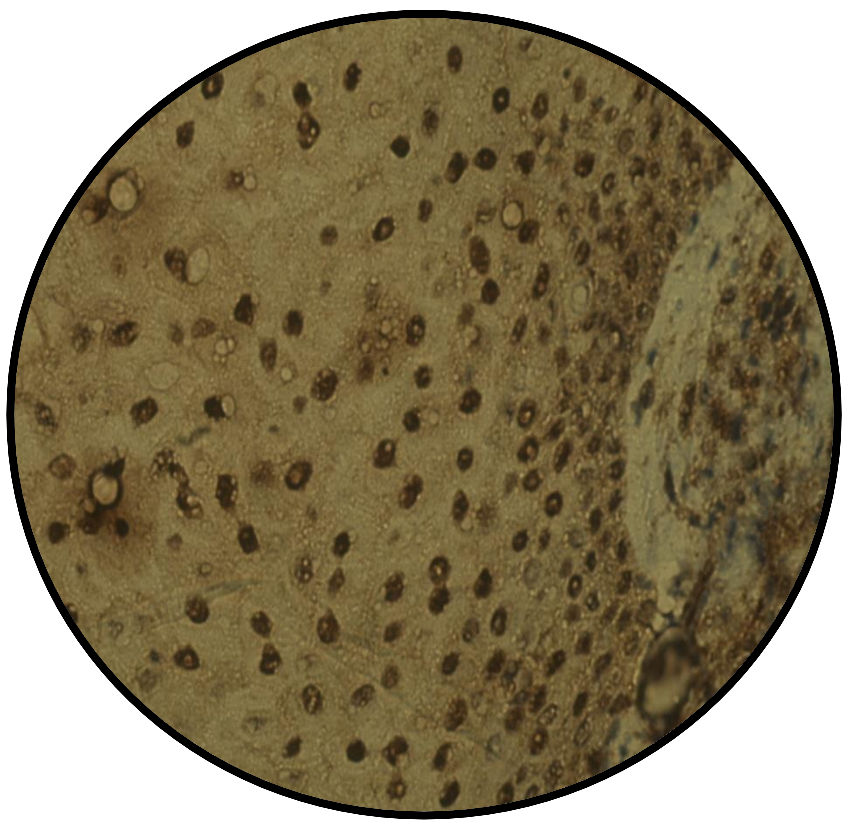

C21

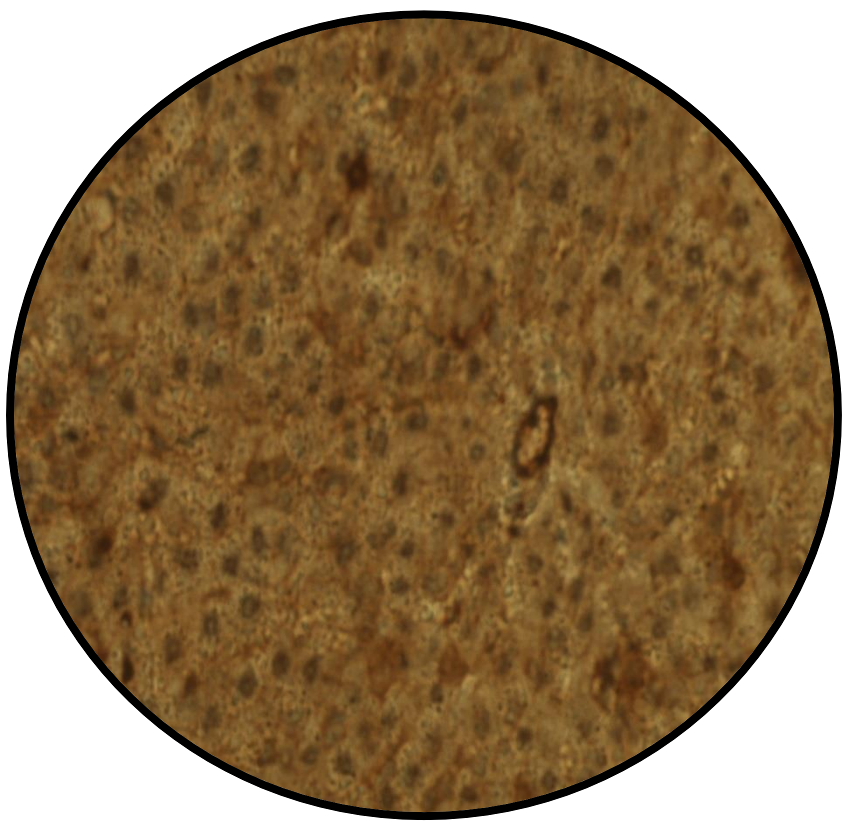

C22

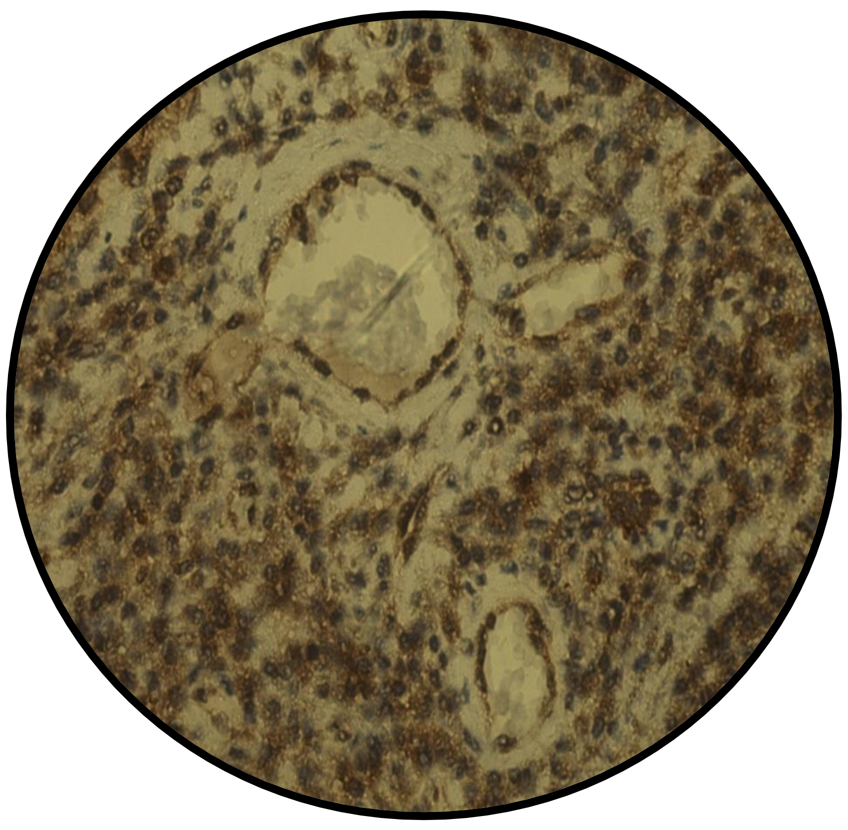

C23

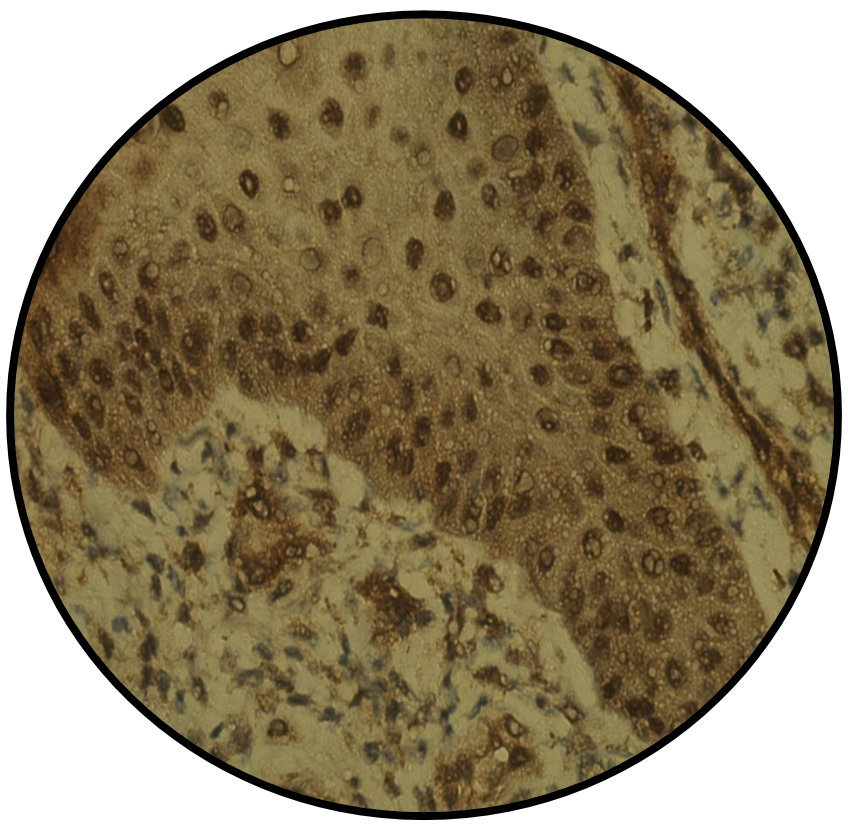

C24

Controls

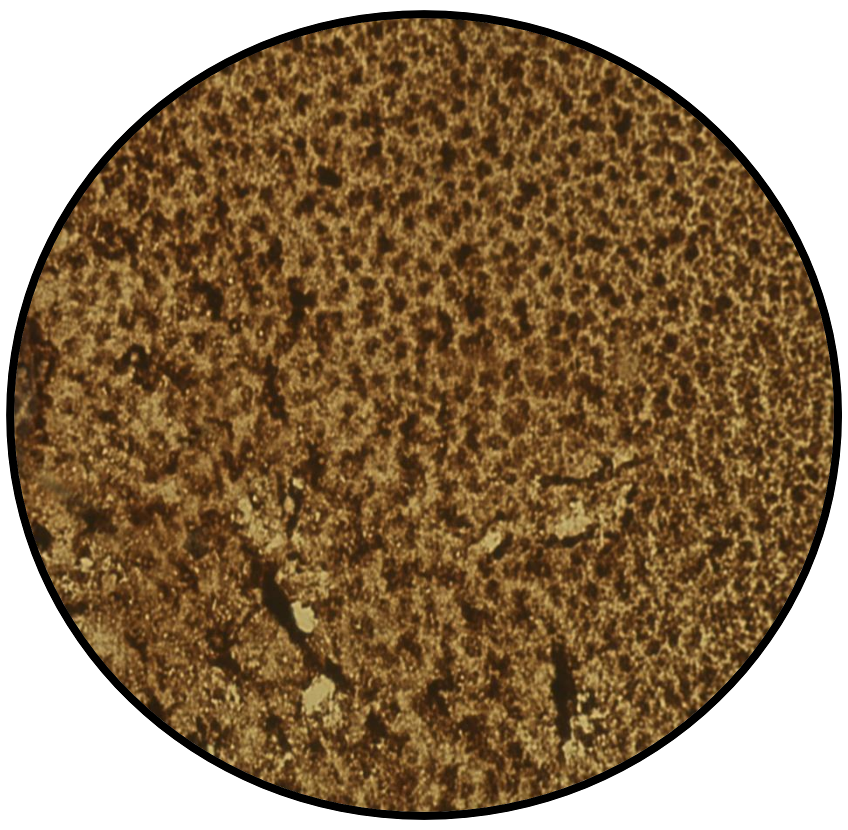
C25

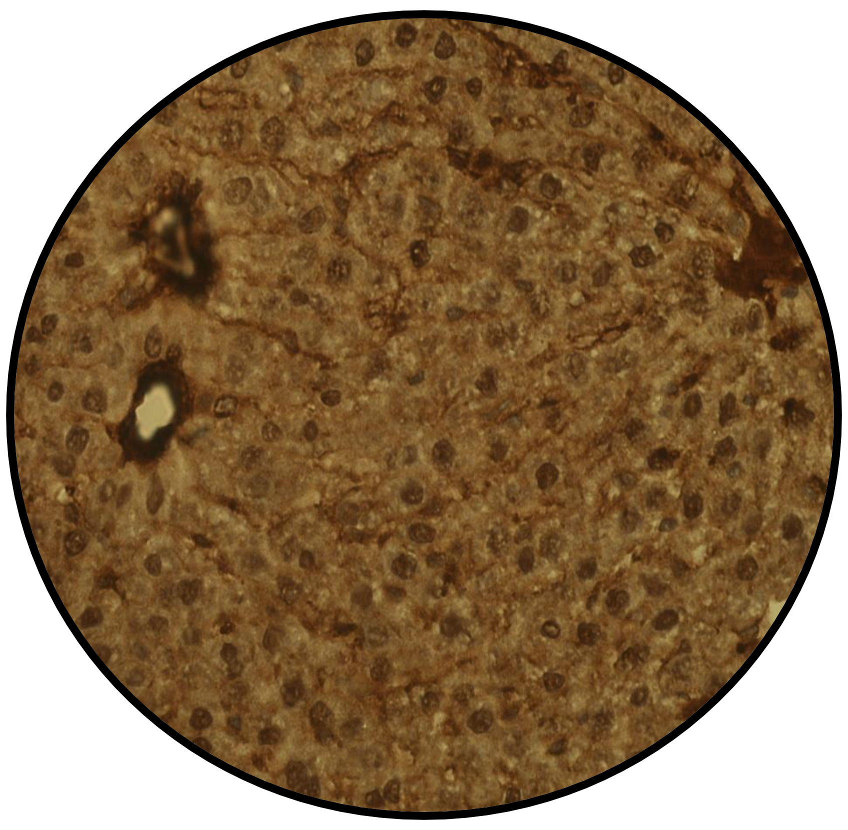
C26

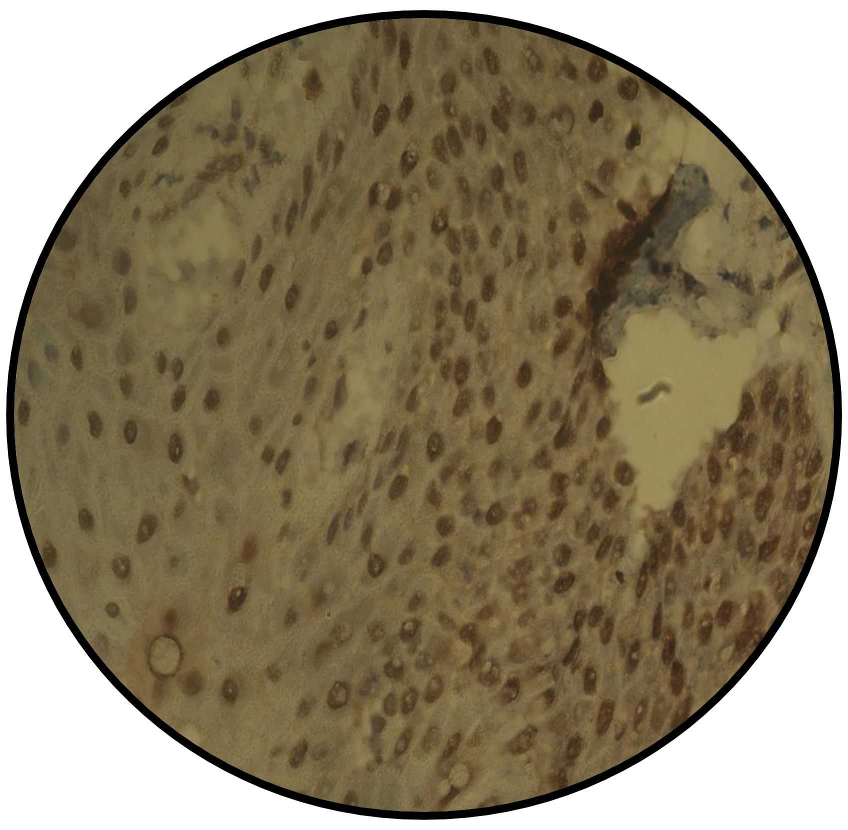
C27

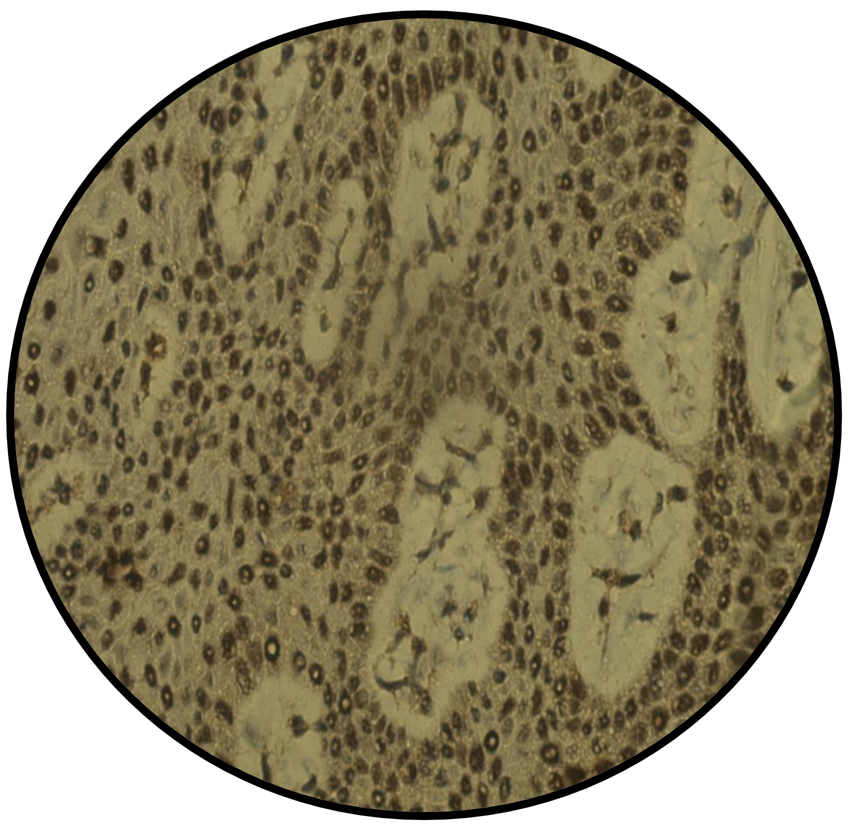
C28

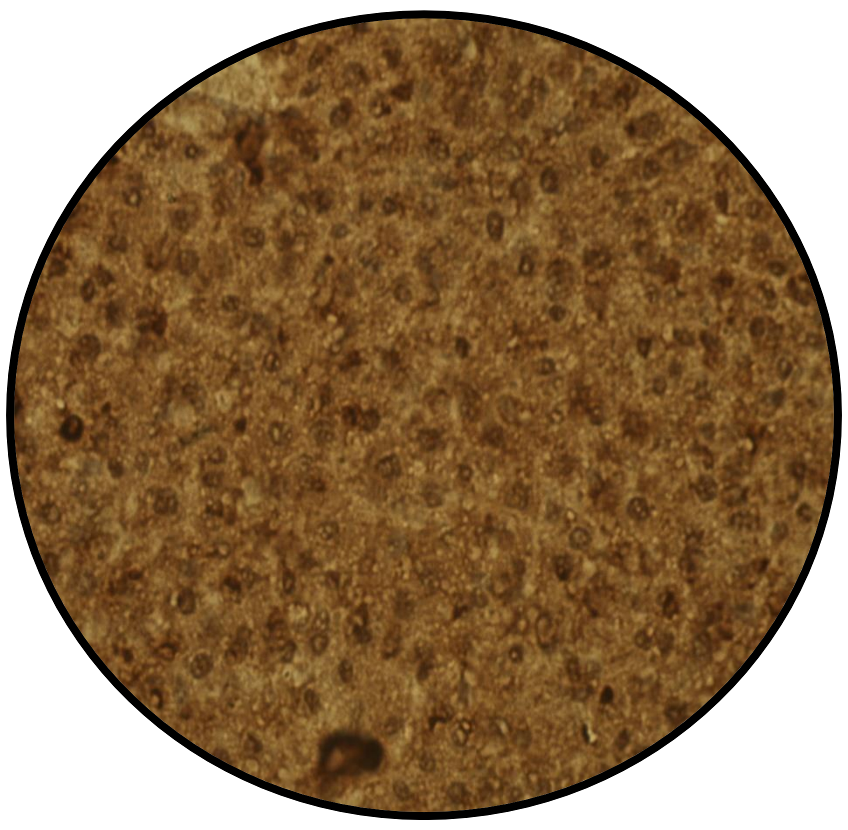
C29

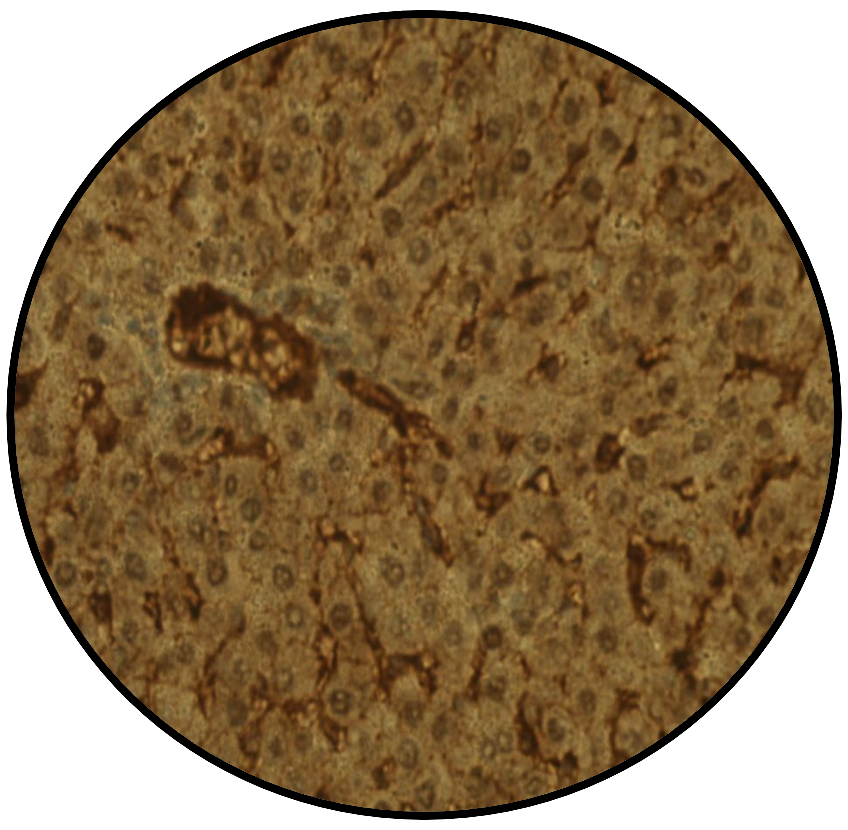
C30

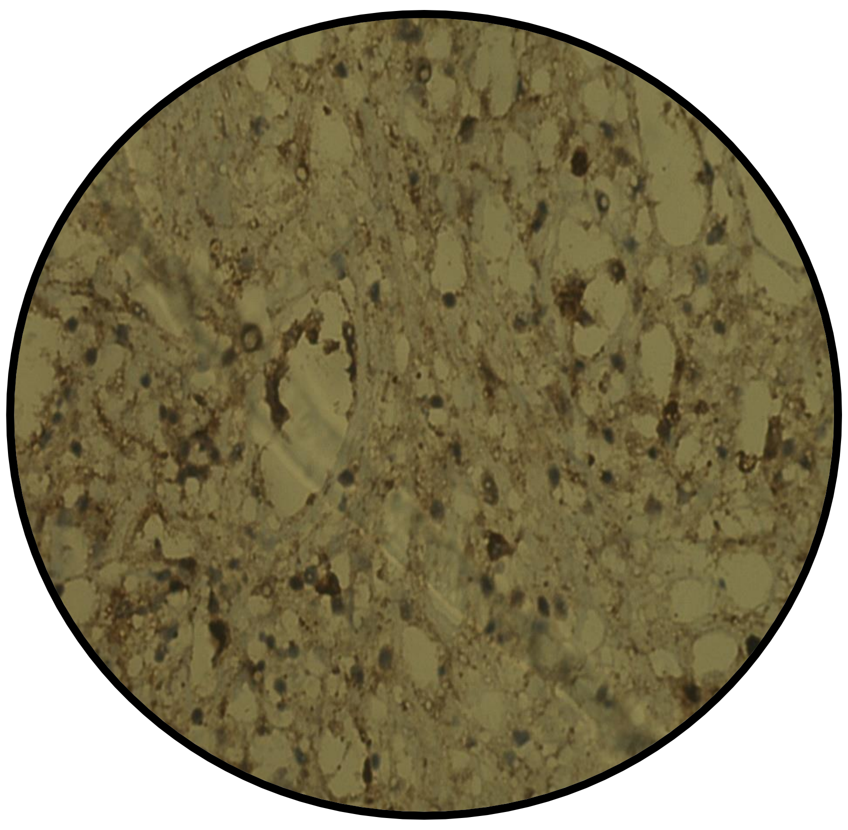
C31

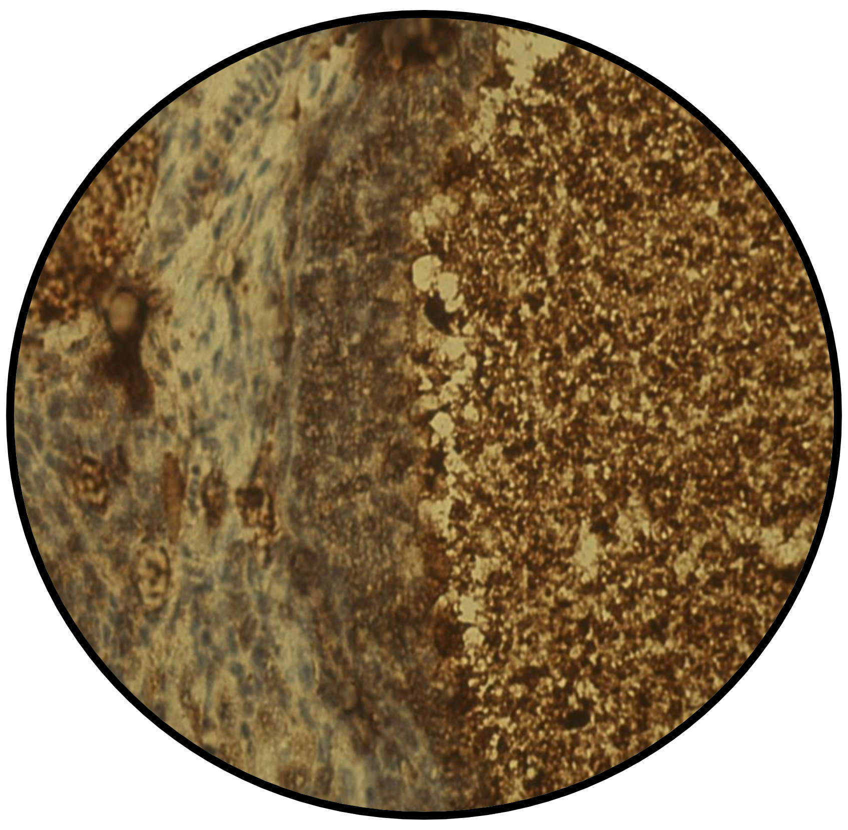
C32

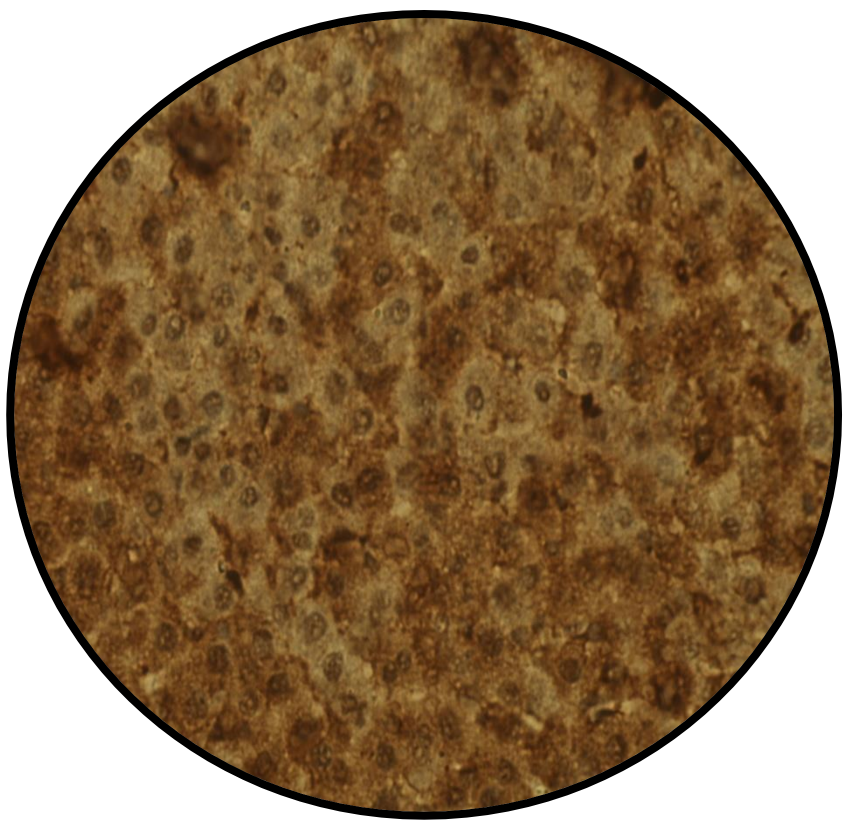
C33

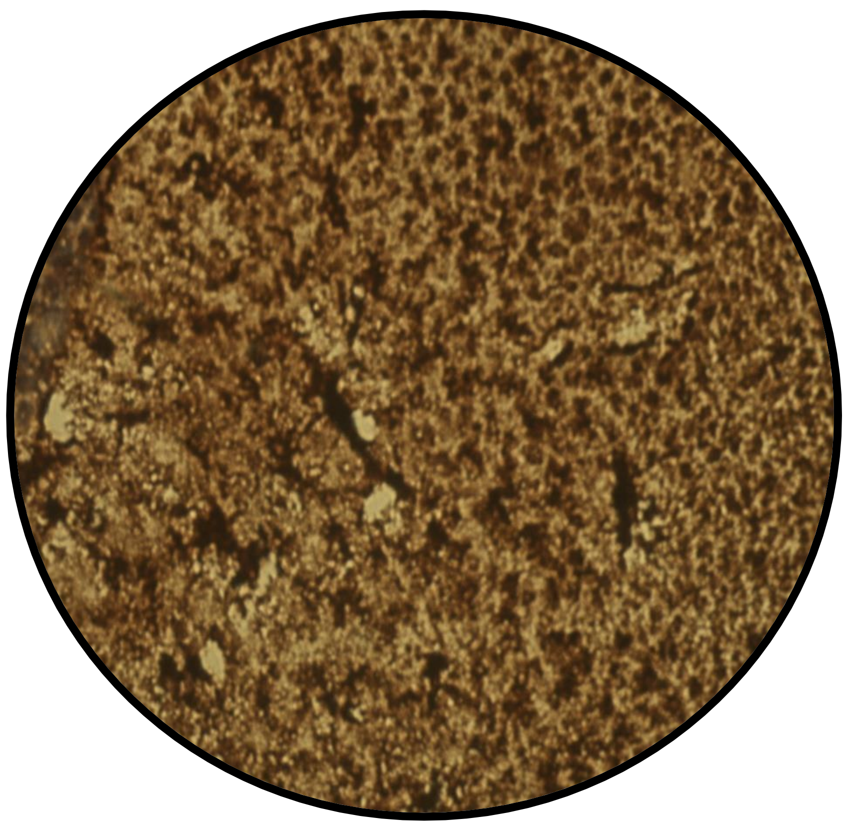
C34

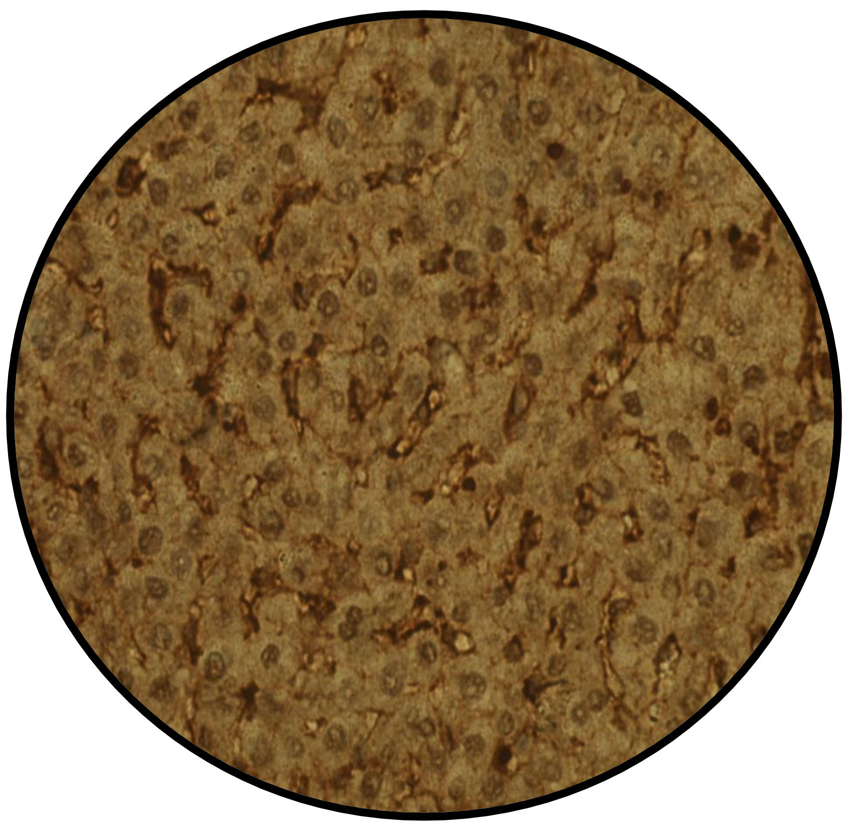
C35

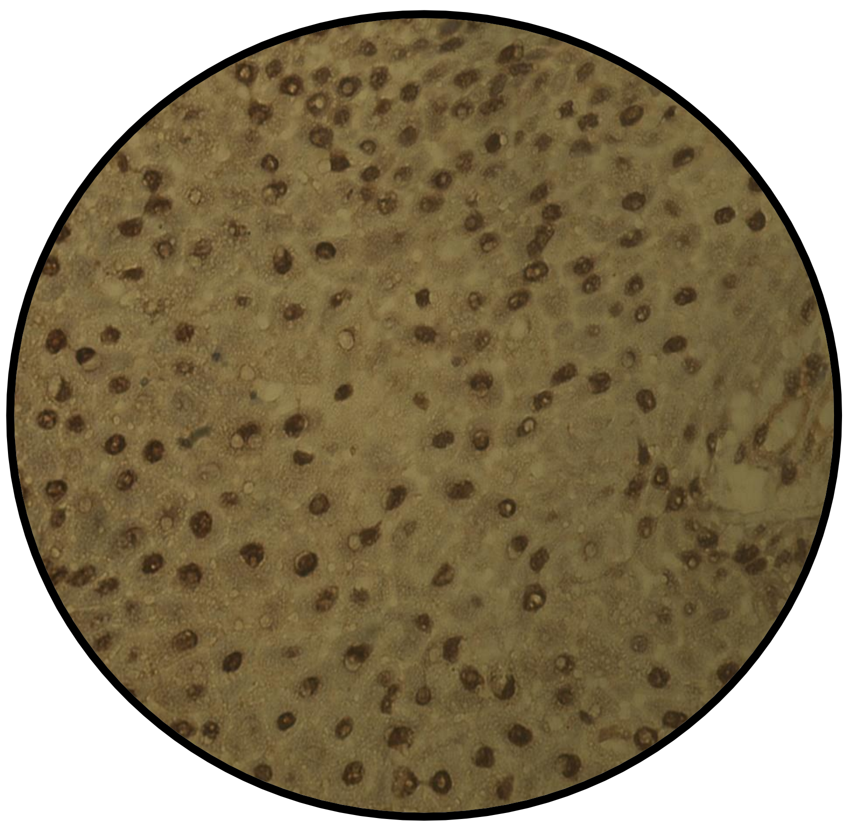
C36

Controls

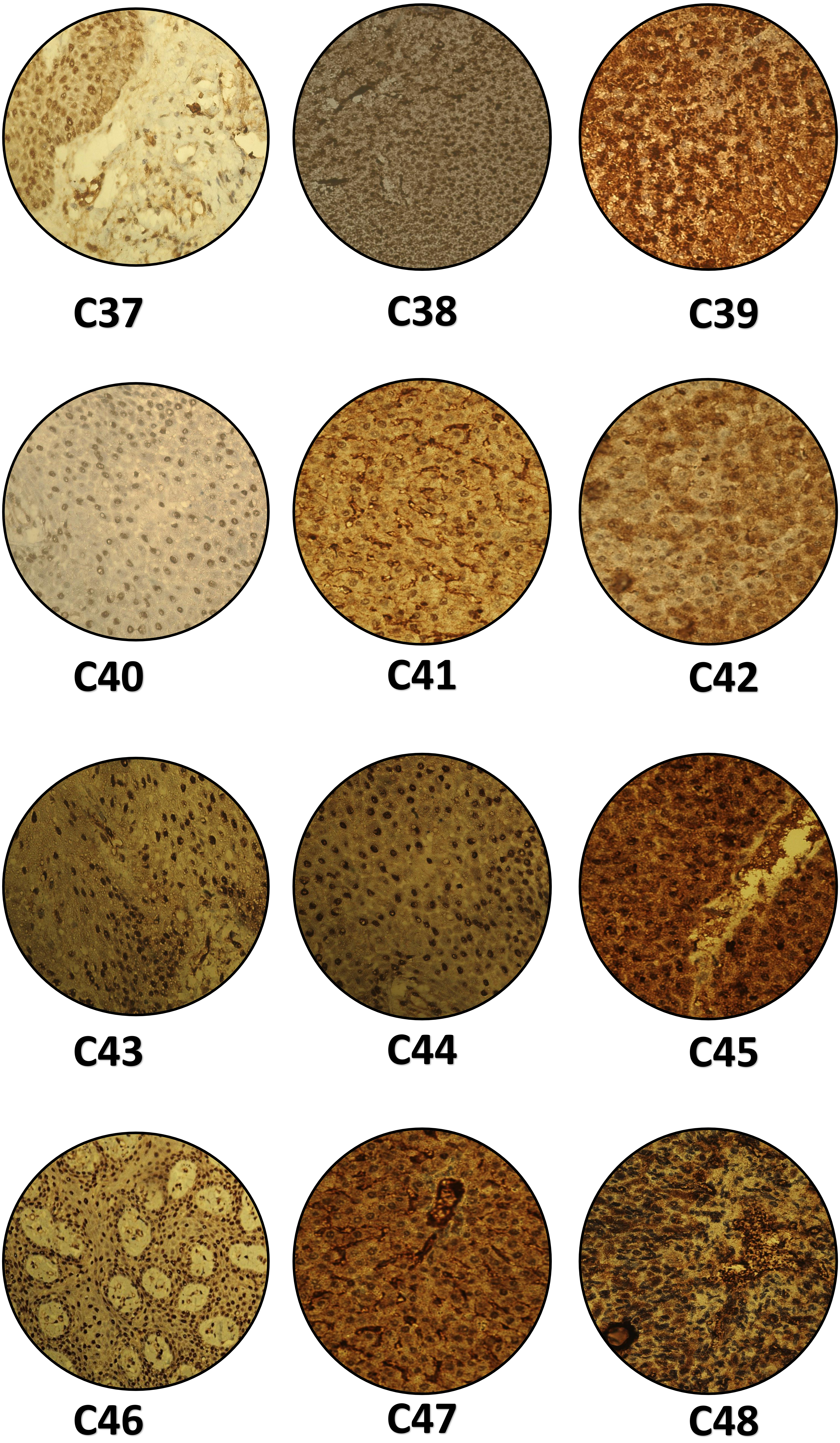

Controls

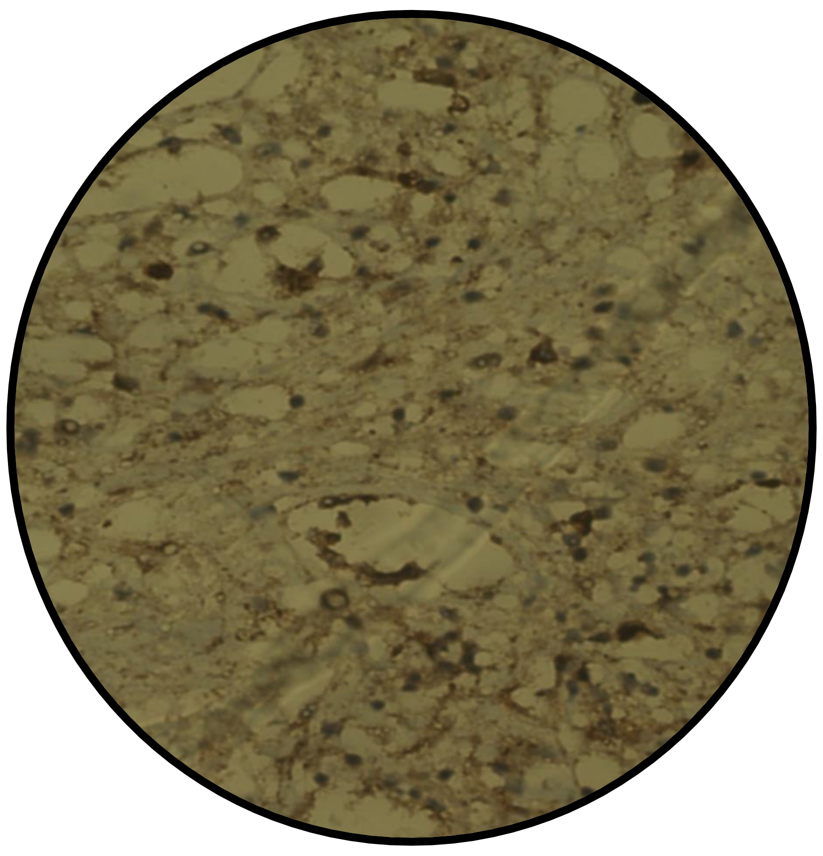

C49

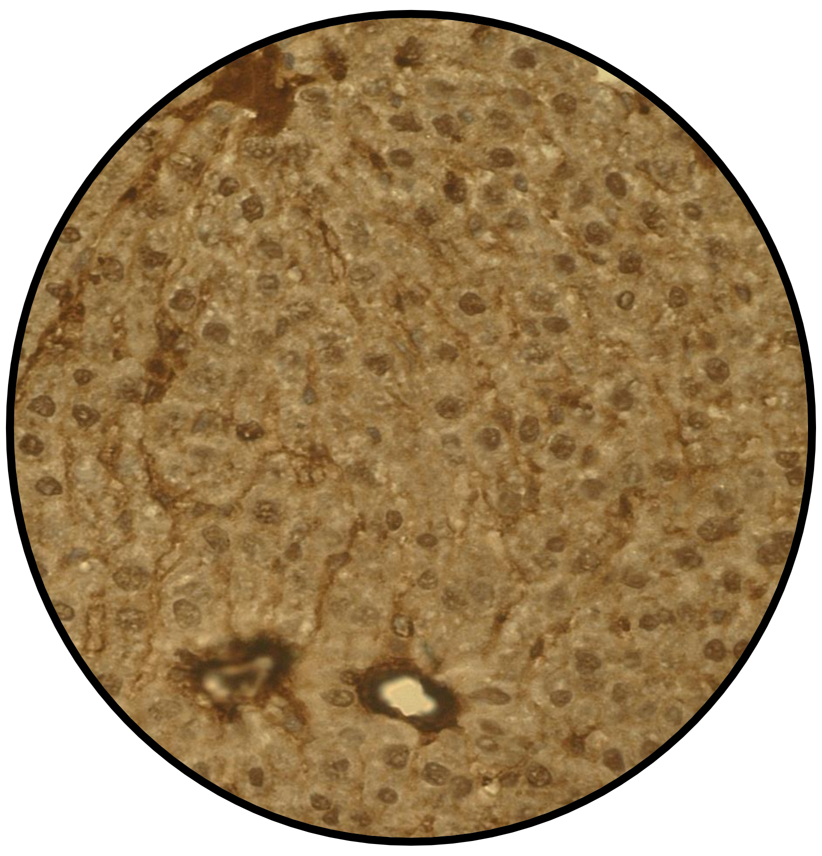

C50

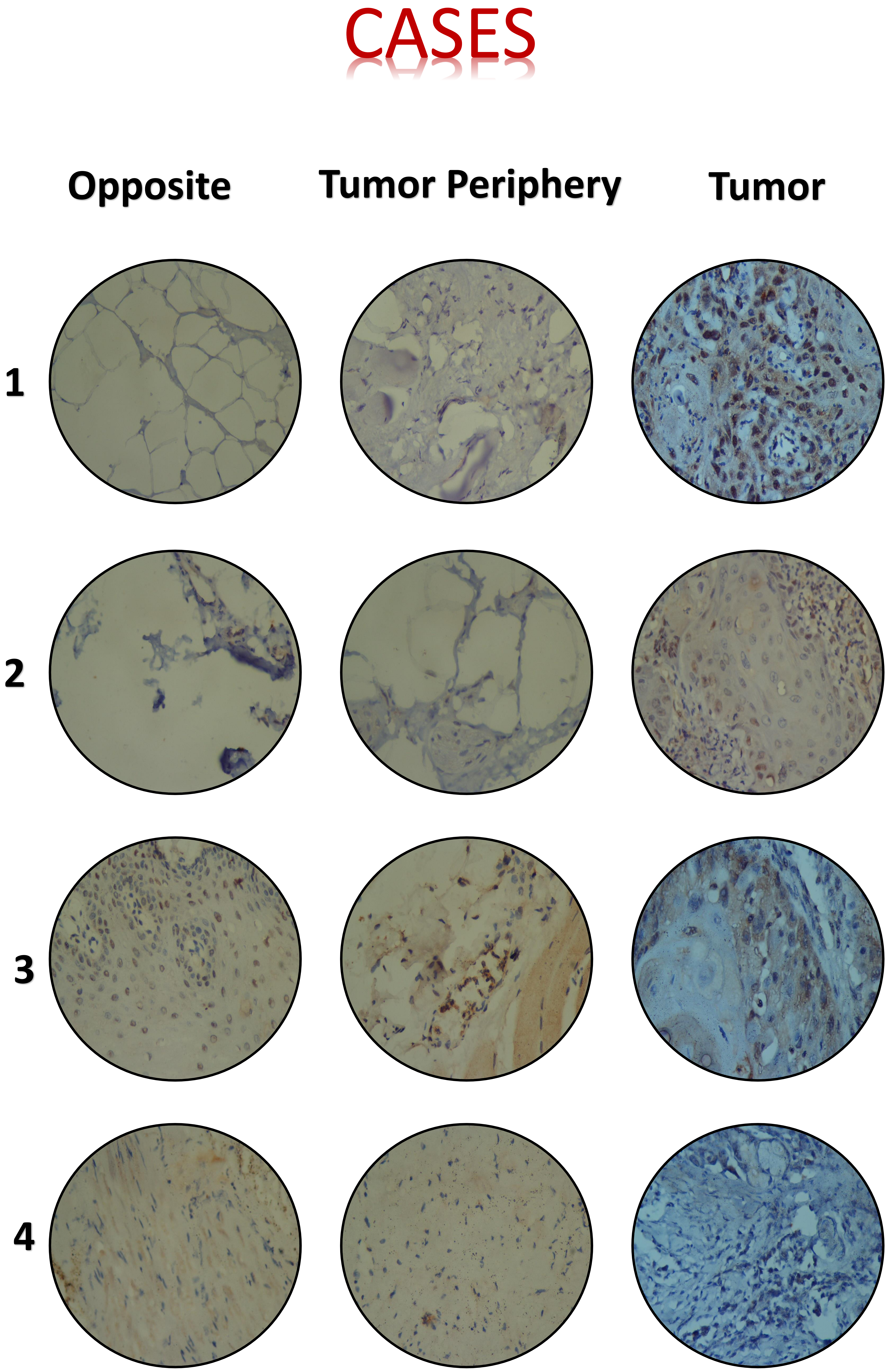
CASES
Opposite
Tumor Periphery
Tumor
1
2
3
4

CASES

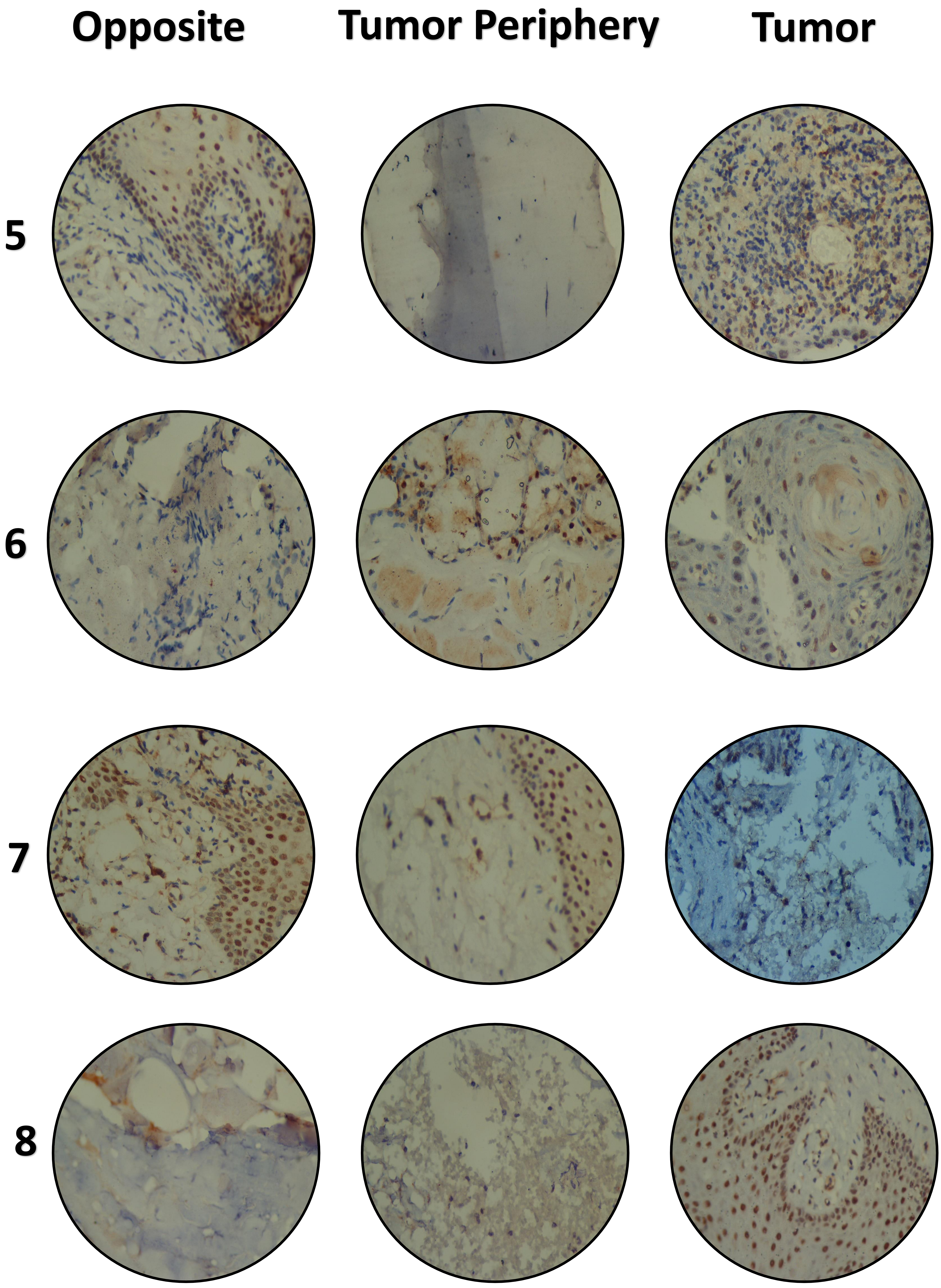

CASES

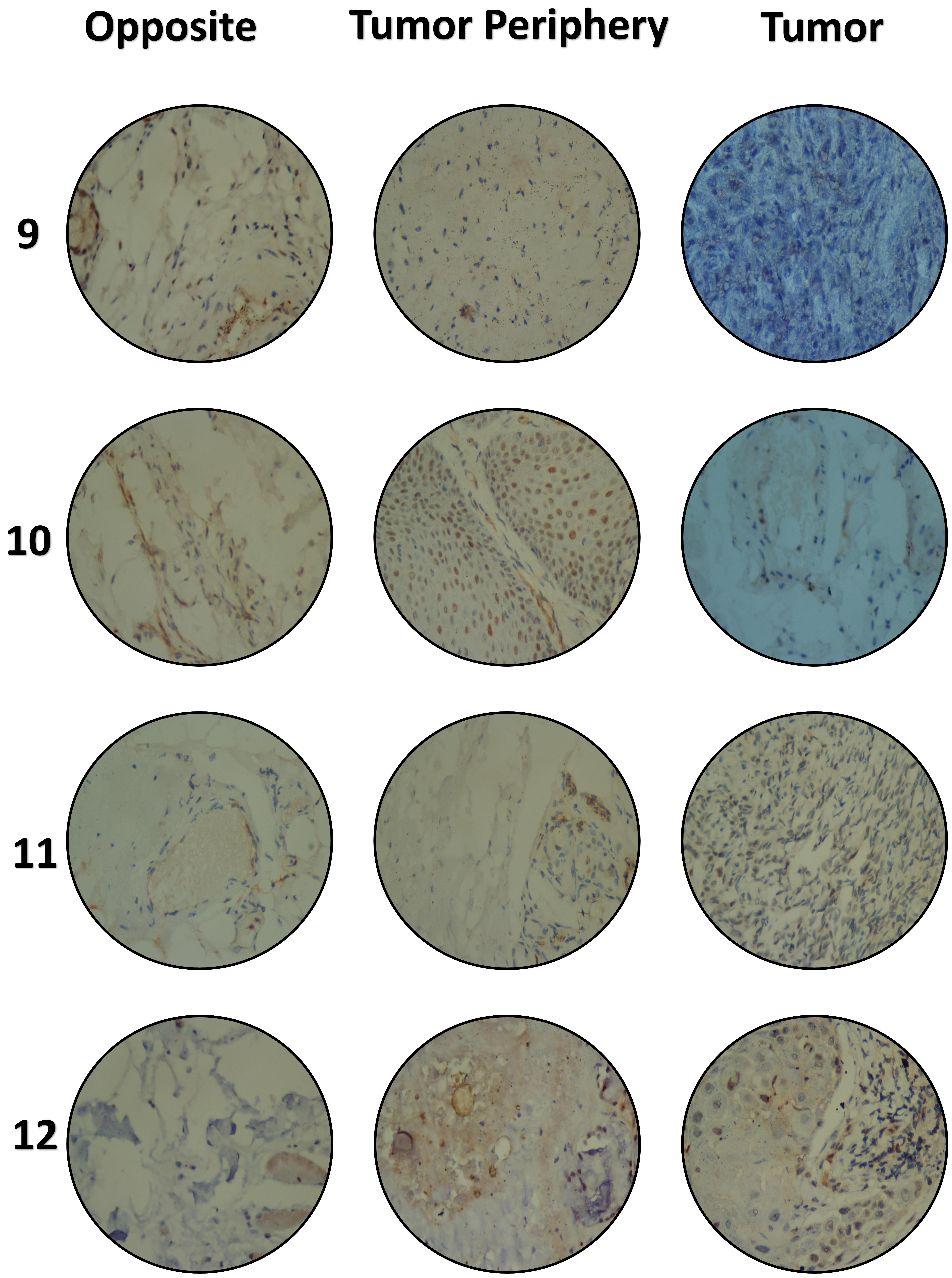

CASES

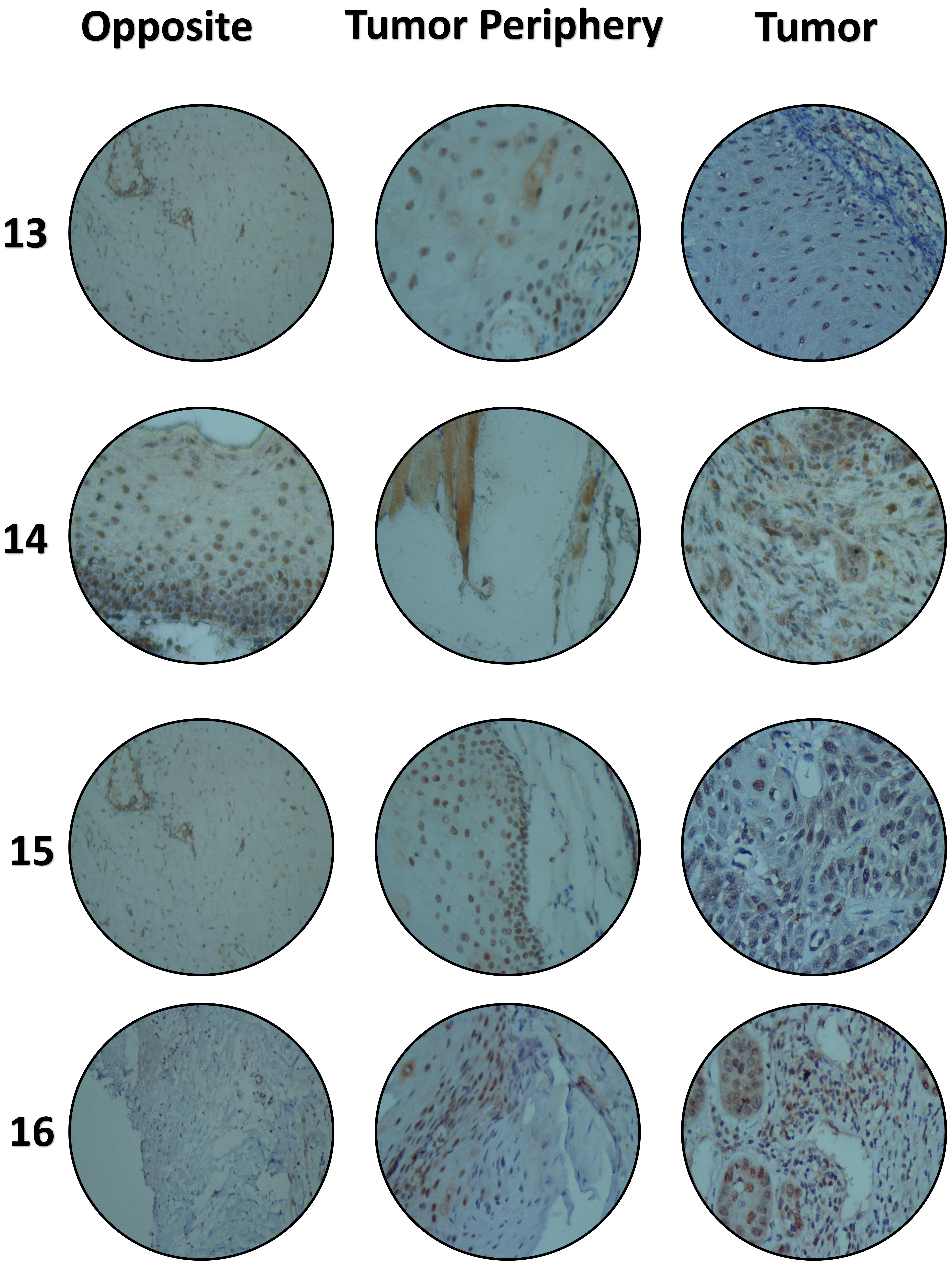

CASES

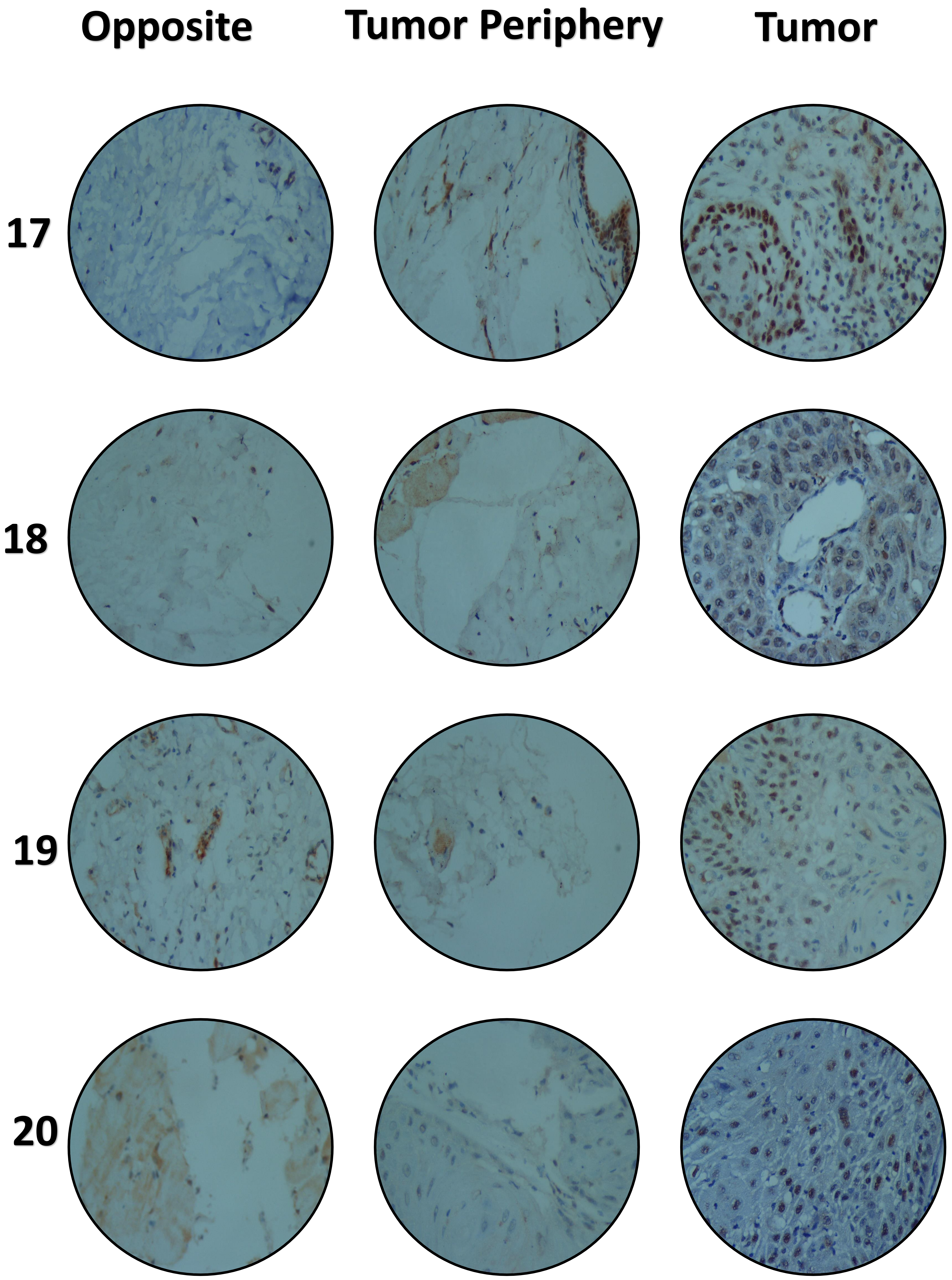

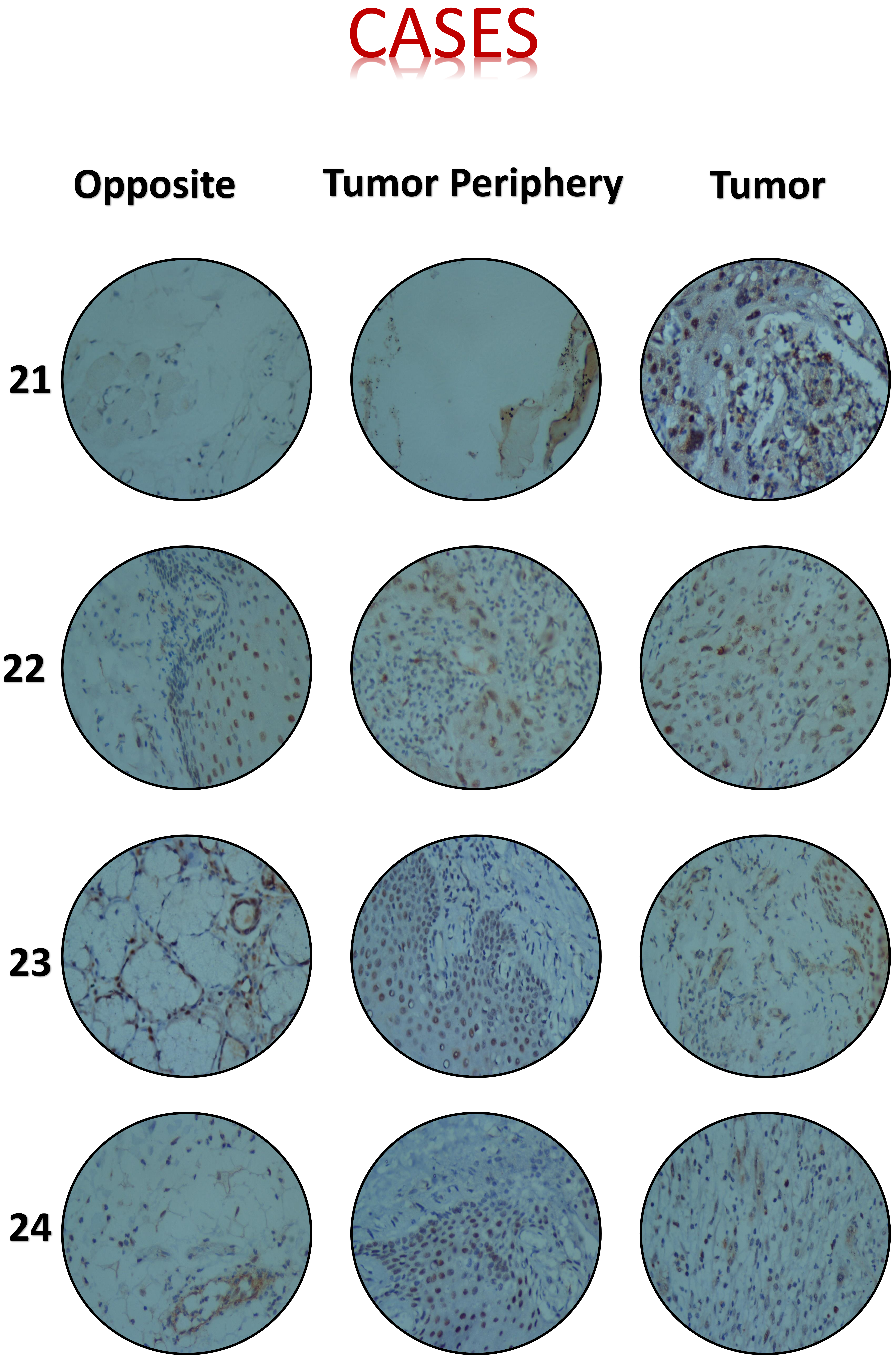
CASES
Opposite
Tumor Periphery
Tumor
21
22
23
24

CASES

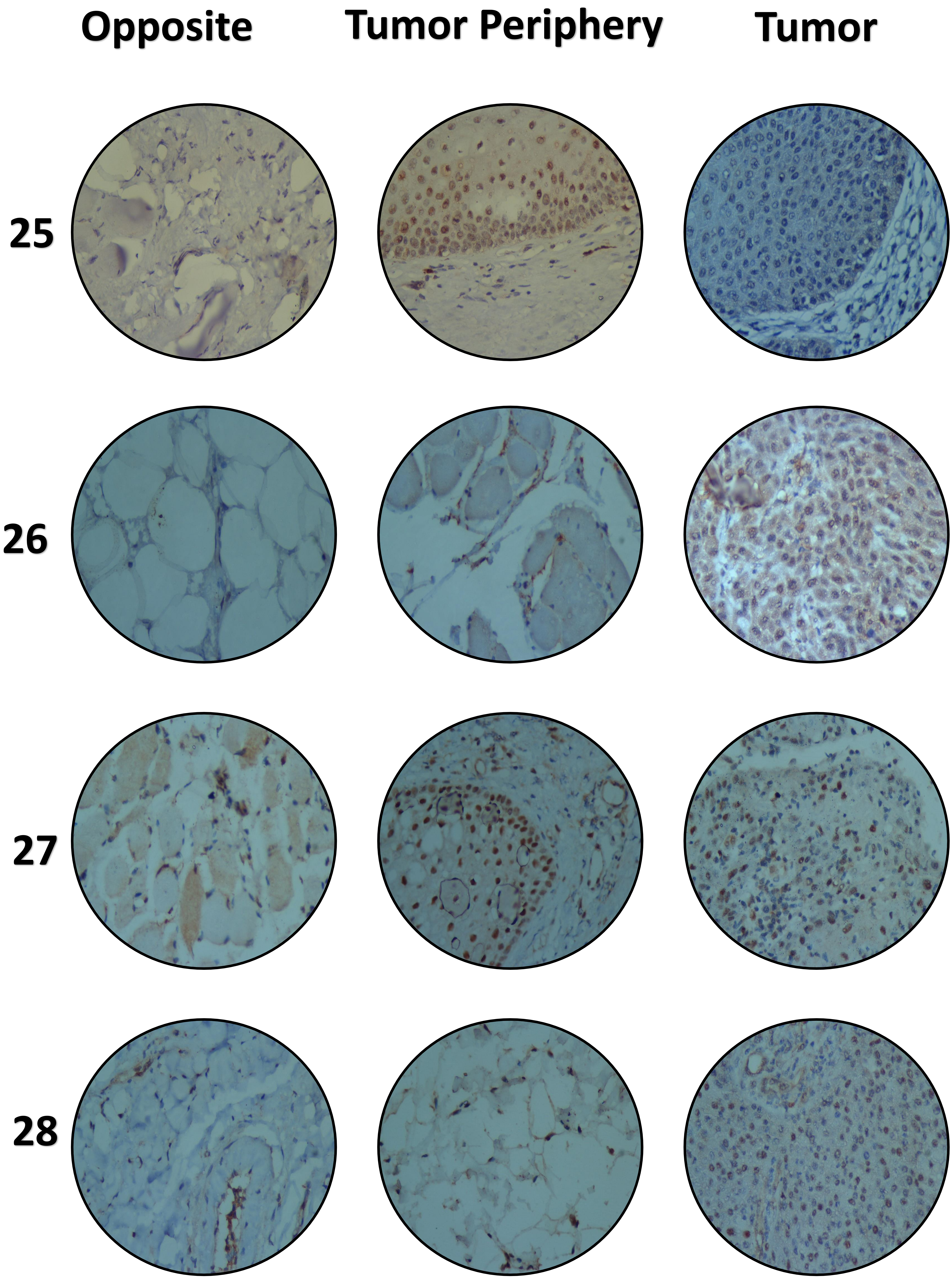

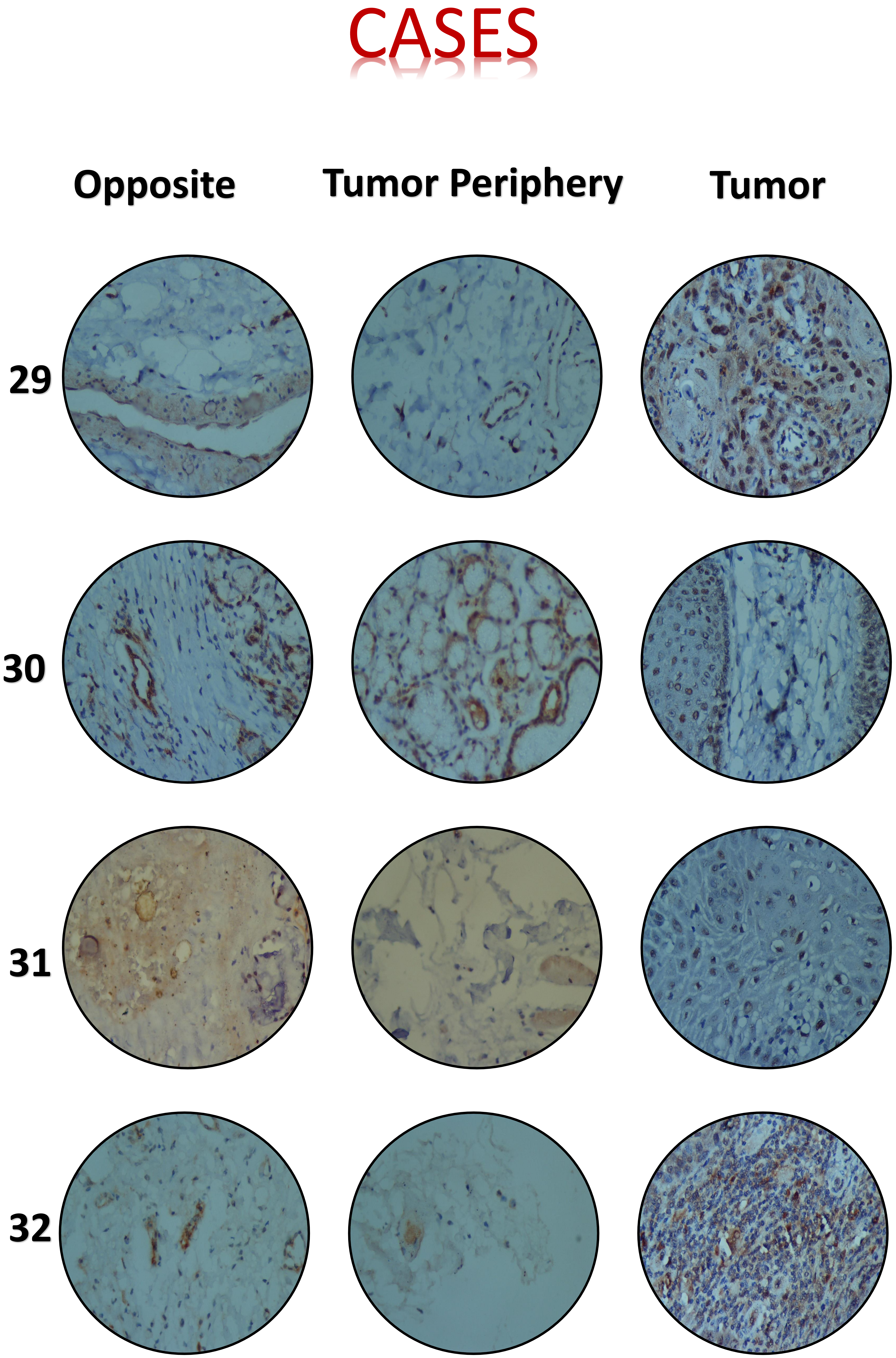
CASES
Opposite
Tumor Periphery
Tumor
29
30
31
32

CASES

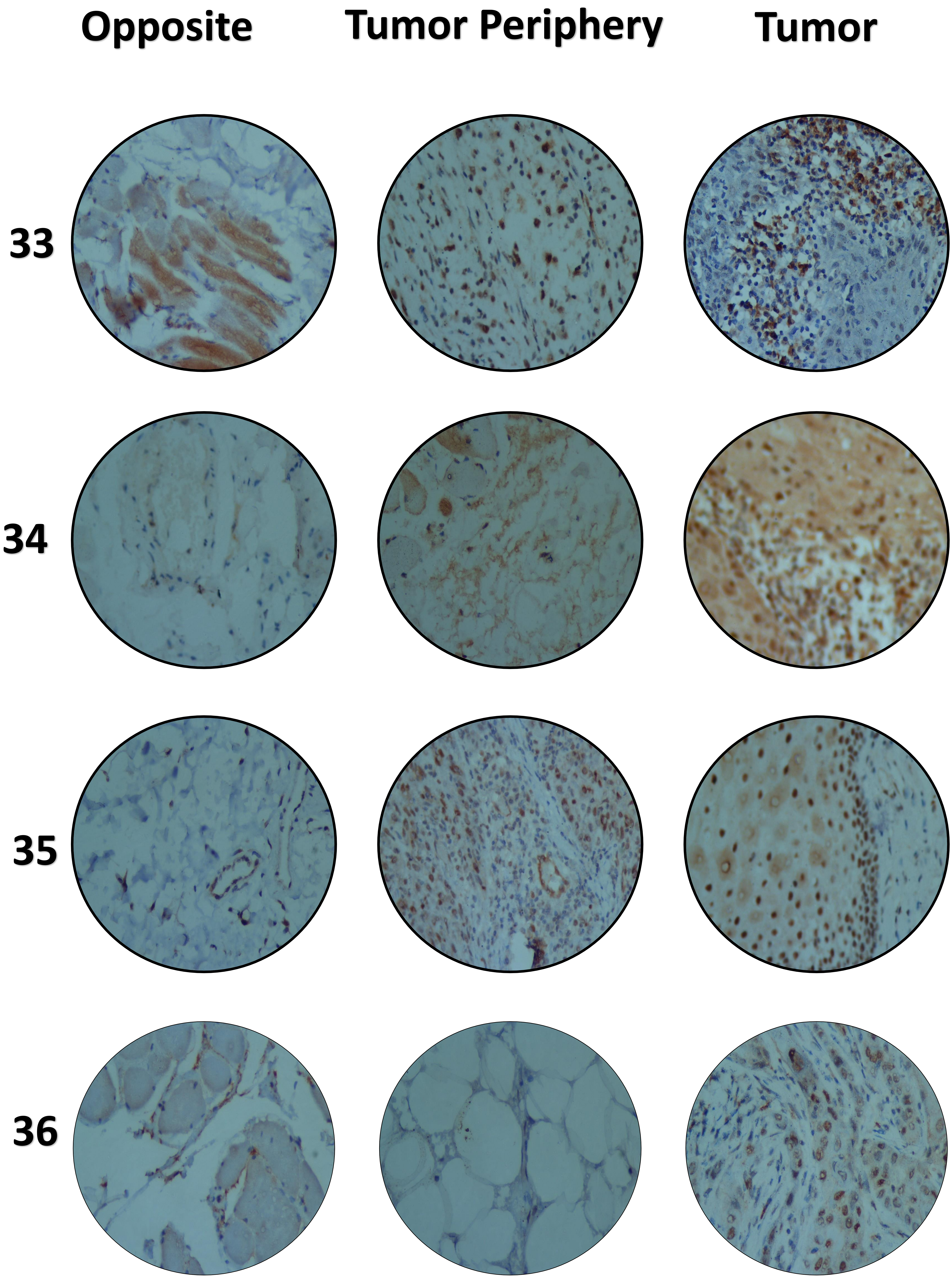

CASES

Opposite | **Tumor Periphery** | **Tumor**

37

38

39

40

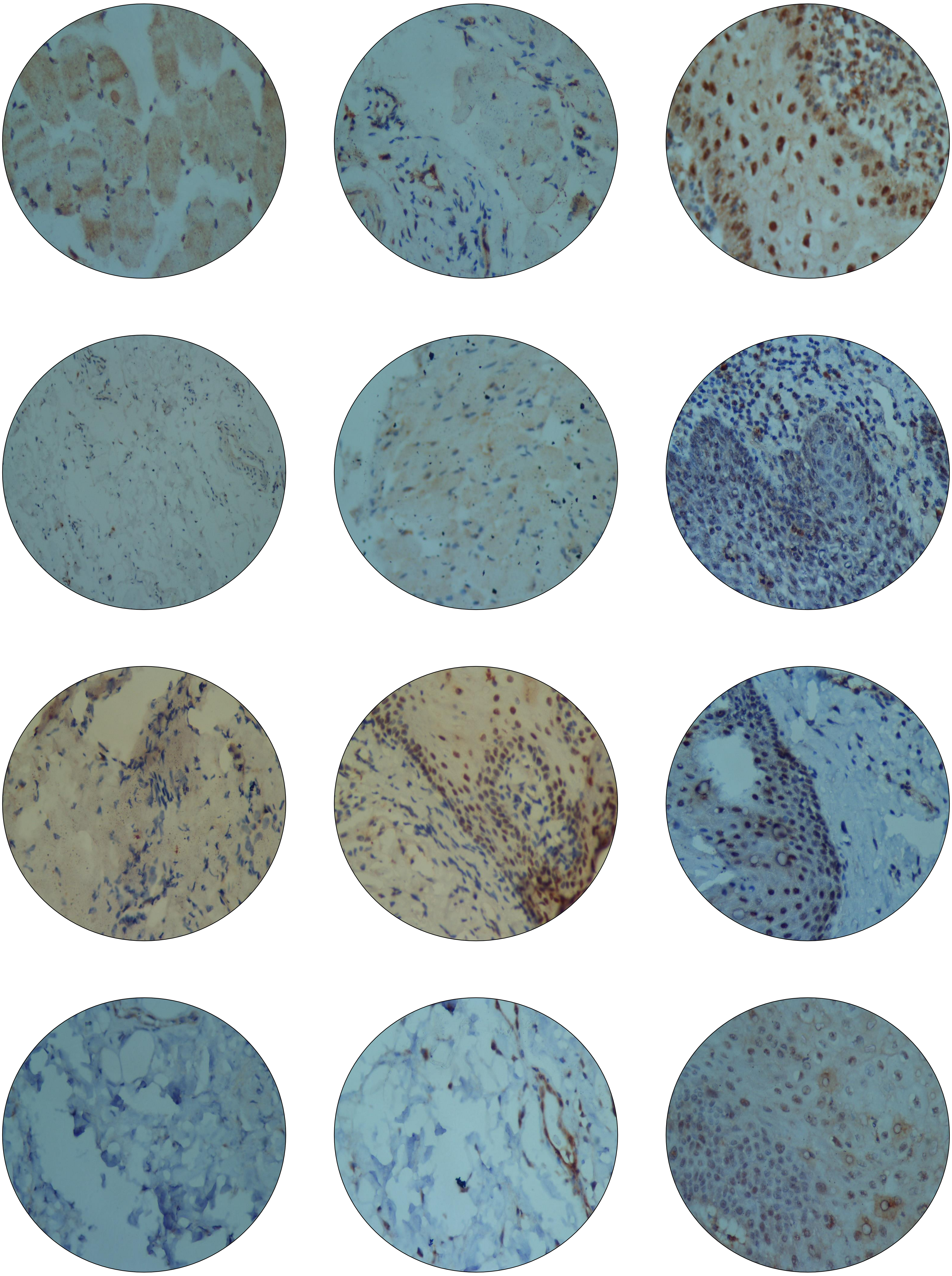

CASES

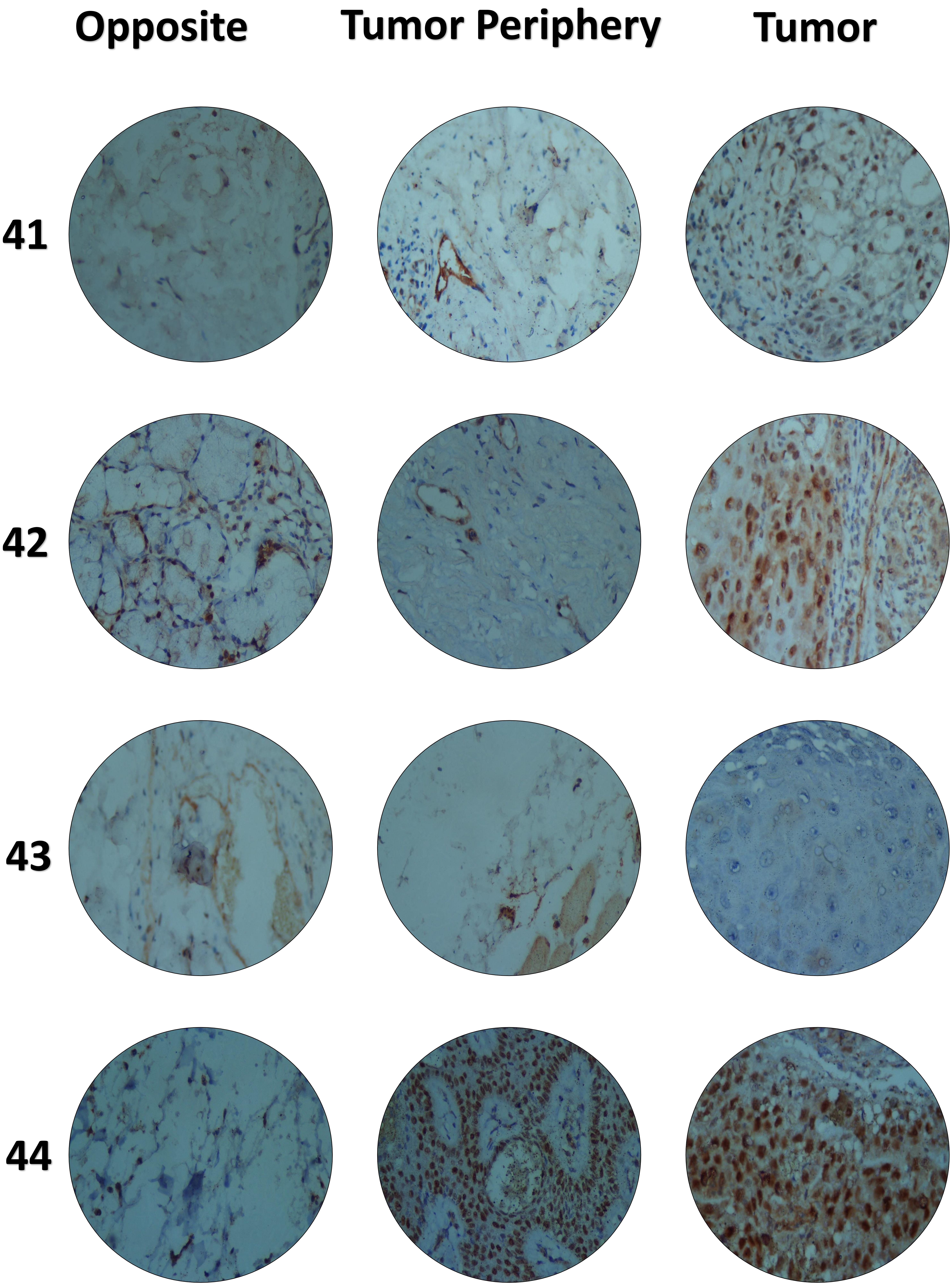

CASES

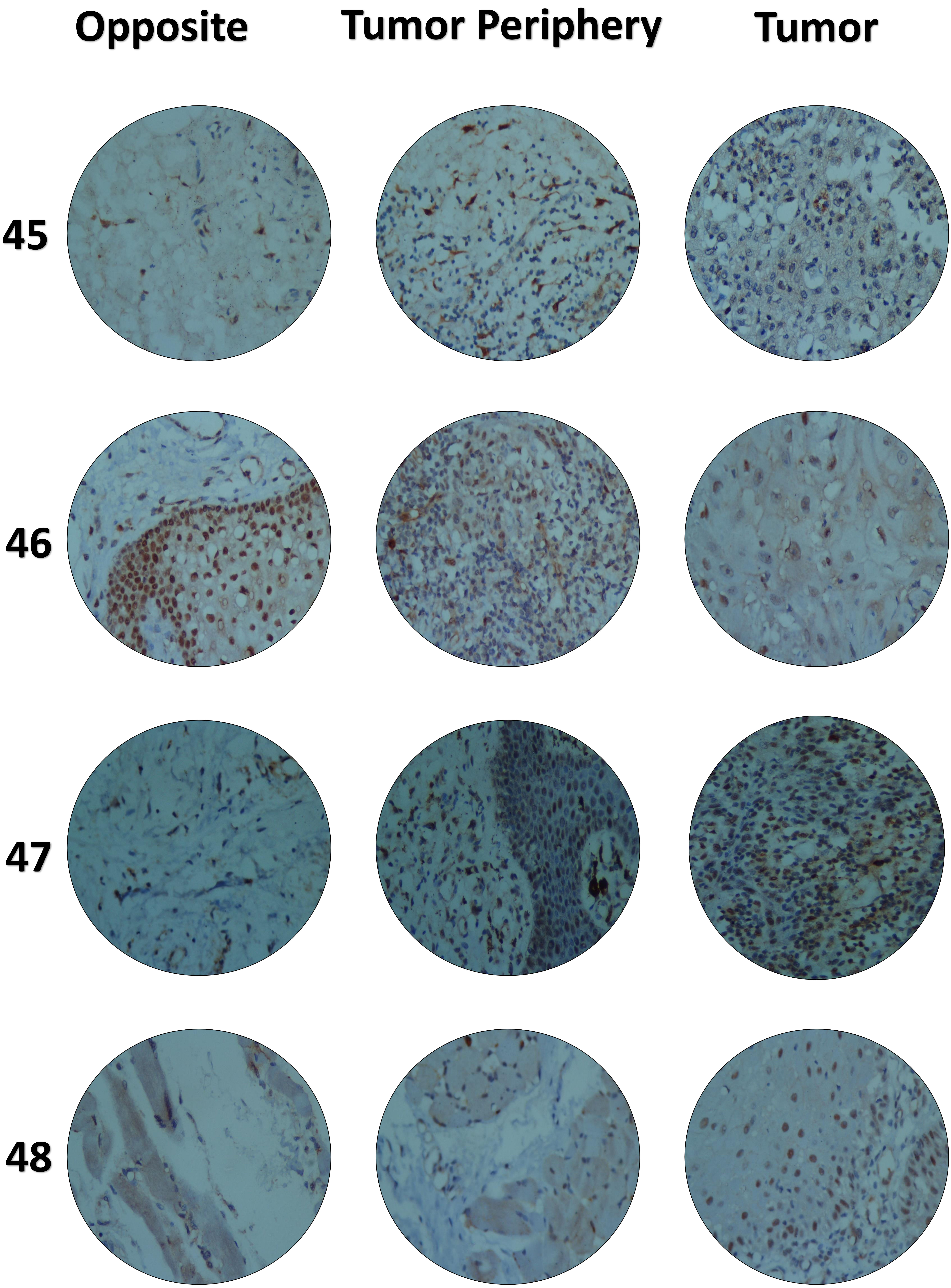

CASES

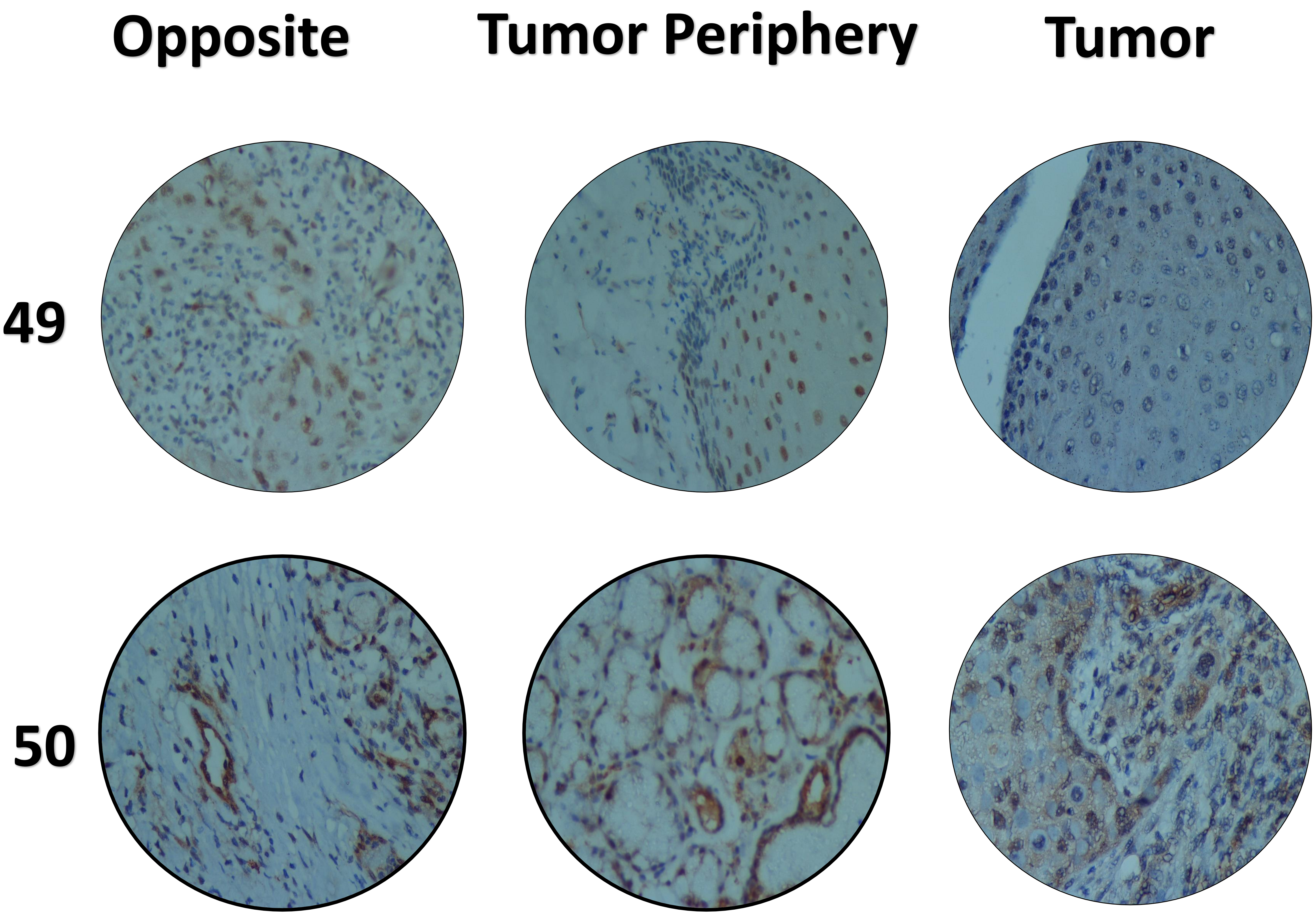

Supplement: Supplementary file 1 — Additional file 1. [file 12885_2022_10014_MOESM1_ESM.zip › IHC control and Kaiso.pdf]
