# Supplementary material for: Expressional variations of Kaiso: an association with pathological characteristics and field cancerization of OSCC
Source: BMC Cancer. 2022 Sep 17;22:990. doi: 10.1186/s12885-022-10014-7 (PMC9482199; doi:10.1186/s12885-022-10014-7)
Supplement: Supplementary file 1 — Additional file 1. [file 12885_2022_10014_MOESM1_ESM.zip › results as advised by reviewer 2.docx]

# Results Re-analysis as Per Request of the Reviewer #2

**Figure 5:** **Differential Expression of Kaiso HP(a) vs P+LP(b) in OSCC and Controls.** The graph in figure **(a)** demonstrate the difference of expression of High Positive values of Expression Kaiso among tissue specimens taken from OSCC cases and controls; whereas figure **(b)** demonstrates differential expression between the Controls and OSCC tissue specimens in terms of Positive and Low Positive values of expressionThe large central line represents mean, whereas small horizontal lines above and below represent the standard deviation.

**Figure 6. Differential Expression of Kaiso in Different Tumor Sizes between High Positive(HP) Expression values (a) and Positive + Low Positive (P+LP)Expression Values (b).** The graph in figure **(a)** demonstrate the difference of expression in Kaiso among different Tumor Sizes in terms of HP; whereas figure **(b)** demonstrates differential expression of Kaiso among different tumor sizes in terms of P+LP.). The large central line represents mean, whereas small horizontal lines above and below represent the standard deviation.

**Figure 7**. **Differential Expression of Kaiso in Different Tumor Grades between High Positive(HP) Expression values (a) and Positive + Low Positive (P+LP)Expression Values (b)**. The large central line represents mean, whereas small horizontal lines above and below represent the standard deviation

**Figure 8. Correlation between Expression of Kaiso (HP) and Histological Feature Scores from Bryne’s TIF Grading System.** The graphs here demonstrate the correlation between expression of Kaiso in OSCC as High Positive Signals and scores given to each histological feature according to Bryne’s scoring system. Each histological feature is correlated separately followed by the correlation with the total malignancy score.

**Figure 9. Correlation between Expression of Kaiso (P+LP) and Histological Feature Scores from Bryne’s TIF Grading System.** The graphs here demonstrate the correlation between expression of Kaiso in OSCC as Positive and Low Positive Signals and scores given to each histological feature according to Bryne’s scoring system. Each histological feature is correlated separately followed by the correlation with the total malignancy score.

**Figure10. Correlation between Expression of Kaiso with Tumor Positive Lymphnodes**. The graphs here demonstrate expression of Kaiso in OSCC as High Positive Signals (HP) (a) and Positve(P)+Low Positive Signals(LP) (b), correlated with number of tumor positive lymphnodes.

**Figure11. Correlation between Expression of Kaiso with Tumor Budding Score**. The graphs here demonstrate expression of Kaiso in OSCC as High Positive Signals (HP) (a) and Positve(P)+Low Positive Signals(LP) (b), correlated with number of tumor budding score.

**Figure12. Correlation between Expression of Kaiso with Tumor Depth**. The graphs here demonstrate expression of Kaiso in OSCC as High Positive Signals (HP) (a) and Positve(P)+Low Positive Signals(LP) (b), correlated with of tumor depth in cm.
